# Whole-genome sequencing of artificial single-nucleotide variants induced by DNA degradation in biological crime scene traces

**DOI:** 10.1007/s00414-022-02911-0

**Published:** 2022-11-10

**Authors:** Kristina Schulze Johann, Hannah Bauer, Peter Wiegand, Heidi Pfeiffer, Marielle Vennemann

**Affiliations:** 1 Institute of Legal Medicine, University of Münster, Röntgenstr. 23, 48149 Münster, Germany; 2 Institute of Legal Medicine, University of Ulm, Albert-Einstein-Allee 23, 89081 Ulm, Germany

**Keywords:** Whole-genome sequencing, Crime scene trace, Time since deposition, DNA degradation

## Abstract

The aim of this study was to identify artificial single-nucleotide variants (SNVs) in degraded trace DNA samples. In a preliminary study, blood samples were stored for up to 120 days and whole-genome sequencing was performed using the Snakemake workflow dna-seq-gatk-variant-calling to identify positions that vary between the time point 0 sample and the aged samples. In a follow-up study on blood and saliva samples stored under humid and dry conditions, potential marker candidates for the estimation of the age of a blood stain (= time since deposition) were identified. Both studies show that a general decrease in the mean fragment size of the libraries over time was observed, presumably due to the formation of abasic sites during DNA degradation which are more susceptible to strand breaks by mechanical shearing of DNA. Unsurprisingly, an increase in the number of failed genotype calls (no coverage) was detected over time. Both studies indicated the presence of artificial SNVs with the majority of changes happening at guanine and cytosine positions. This confirms previous studies and can be explained by depurination through hydrolytic attacks which more likely deplete guanine while deamination leads to cytosine to thymine variants. Even complete genotype switches from homozygote 0/0 genotypes to the opposite 1/1 genotypes were observed. While positions with such drastic changes might provide suitable candidate markers for estimating short-term time since deposition (TsD), 11 markers were identified which show a slower gradual change of the relative abundance of the artificial variant in both blood and saliva samples, irrespective of storage conditions.

**Supplementary Information:**

The online version contains supplementary material available at 10.1007/s00414-022-02911-0.

## Introduction

Forensic DNA profiling has become a routinely used tool in analyzing forensic evidence. Autosomal short tandem repeat (STR) markers are utilized to generate DNA profiles that may identify a person of interest or even link them to the scene of a crime. One major challenge analysts face when working with biological material is DNA degradation as it limits the correct interpretation of STR typing results. However, DNA degradation might also have its benefits: the degree of degradation has been linked to the age of a biological stain [1] and knowledge of the time since deposition (TsD) of a biological trace found at the scene can be important intelligence for criminal investigation. Knowing whether a trace has been deposited before, after, or at the estimated time point of the crime could help to determine the relevance of a trace to the investigation at hand. This information saves resources by avoiding cost-intensive and time-consuming analyses of irrelevant trace material.

Several studies on the estimation of the age of a biological trace have been published e.g., [2], based on optical detection strategies like visible absorbance spectroscopy [3–7] or the analysis of the presence of hemoglobin derivatives [8–11]. While these methods are highly promising for bloodstains, alternative approaches are needed for other biological materials, and in recent publications the TsD was estimated using molecular methods like quantification of RNA degradation in body fluid-specific markers. Various mRNA and miRNA markers were identified and successfully applied for the estimation of TsD not only in blood, but also in menstrual fluid, saliva, semen, and vaginal secretion samples by analyzing their RNA degradation patterns [12]. Even though robustness is not yet considered to be at the level needed for forensic casework [13, 14], such methods are highly promising for future investigations. More recently, microbes were investigated for estimating the TsD of saliva samples but it was indicated that the high inter-individual variation surpasses the time-dependent variation [1]. Schneider et al. demonstrated the general feasibility of LC–MS-based proteomics to determine the TsD over a period of 2 months and samples stored under two different storage conditions [15].

Any method based on DNA analysis is generally easier incorporated into the standard workflow of forensic genetic laboratories compared to RNA- or protein-based technologies [12, 16, 17]. Overballe-Peterson et al. stated that massively parallel sequencing (MPS) allows further insight into DNA degradation over time [18] because this technique allows the detection of all results of DNA degradation. 

Various external factors such as ultraviolet light, radiation, temperature, or humidity damage DNA structure due to the DNA’s limited chemical stability. One of the main chemical reactions by which DNA is damaged is hydrolytic degradation, i.e., the cleavage of chemical bonds by the addition of water [19–22]. There are two main mechanisms by which hydrolysis attacks DNA integrity, that of base loss from the 2′-deoxyribose backbone and that of deamination. Hydrolysis of the DNA backbone attacks the linkage between the deoxyribose carbon atom and the base, and is the main reason for DNA degradation in dead tissue [23]. When the DNA bases cytosine (C), adenine (A), and guanine (G) undergo the process of spontaneous deamination, they will be converted to uracil, hypoxanthine, and xanthine, respectively, and ammonia. Deamination of DNA bases leads to structure instability and sequence variation: xanthine is known to base pair with both cytosine and thymine (T), while hypoxanthine predominantly pairs with cytosine to promote A to G mutations [24].

While newly developed forensic DNA typing strategies are consistently validated to produce high-quality data from low-quality DNA, information on the exact mode of DNA degradation and their time-dependent characteristics in forensic crime scene traces are poorly studied [25]. Consequently, the aim of our study aimed was to investigate signs of DNA degradation in whole-genome sequencing (WGS) data. Here, we focus on artificial single-nucleotide variants (SNVs) that are products of DNA damage over time. In a preliminary study we show that SNVs are present in blood samples stored for up to 120 days. Based on these observations, a follow-up study was performed to analyze the effect of different storage conditions (dry and humid) and potential differences between body fluids (blood and saliva). Again, three different time points were analyzed in the follow-up study similar to the time points in the preliminary study to confirm changes over a longer storage duration. The follow-up study of saliva and blood samples stored under humid and dry conditions proofs the concept and allows the identification of potential markers worthy of further investigation for their ability to estimate TsD.

## Material and methods

### Samples

Samples were collected with informed consent from one individual only to eliminate interindividual variations caused, i.e., by the general health status of the donor. In a preliminary study, blood samples (*n* = 3) were collected by venipuncture and stored at room temperature (RT) for 0, 21, and 120 days. For the larger-scale follow-up study, a blood sample from one donor was collected by venipuncture and spots of 40 µL of blood were prepared on sterile cellulose pads. Saliva samples (*n* = 5) from the same donor were collected by swabbing the inside of the cheek with a forensic swab. A total of 5 blood samples and 5 saliva samples were stored for 0, 22, and 92 days under humid and dry conditions, respectively. Samples stored under dry conditions were stored in transparent plastic boxes protected from direct UV light. Samples stored under humid conditions were stored in transparent plastic boxes with water-covered base. Time point 0 was used as a positive control.

### DNA extraction and quantification

DNA from blood samples was extracted using the Maxwell® RSC Blood DNA Kit on the Maxwell® 16 Forensic Instrument (Promega, Mannheim, Germany) and eluted in a final volume of 50 µL. Per sample, four spots of 40 µL blood were extracted separately and the eluates were subsequently pooled to a total volume of 200 µL. DNA from saliva samples was extracted using Maxwell® FSC DNA IQ™ Casework Kit (Promega, Mannheim, Germany). Samples were quantified using Promega’s PowerQuant® System using 5 µL of PowerQuant 2 × MM, 0.5 µL of 20 × Primer/Probe/IPC-Mix, 3.5 µL HPLC, and 2 µL of the sample. Prior to NGS library construction, DNA was sheared mechanically to allow analysis of natural strand breaks at positions weakened due to depurination in future analyses of these datasets [18]. Shearing to 250 bp was performed following the manufacturer’s guideline for the Covaris® M220 Focused-ultrasonicator (Covaris, Woburn, MA, USA) with a duty cycle of 10%, intensity 4, and 200 cycles per burst for 80 s. After shearing, the DNA was quality checked by 2100 Bioanalyzer Instrument with High Sensitivity DNA Kit (Agilent, Waldbronn).

### Library preparation and whole-genome sequencing

Libraries were prepared using the NEBNext Ultra II DNA Library Prep Kit according to manufacturer’s recommendations (New England Biolabs). Library quality was checked by a TapeStation using D1000 ScreenTape with the TapeStation Analysis Software 3.1.1. The initial three blood samples of the preliminary study were sequenced on the NextSeq 500 System (Illumina, CA, USA) using a high-output of 300 cycles (2 × 150 cycl., 20 × coverage). The samples for the follow-up study were sequenced on Illumina’s NovaSeq 6000 System with a S4 flow cell (2 × 150 cycl., 30 × coverage). Sequencing was performed at Genomics & Transcriptomics Laboratory (GTL) at Heinrich-Heine-University Düsseldorf.

### Bioinformatics and data analysis

Raw data analysis consisted of quality control and paired-end read alignment to the GRCh38 reference with Burrows-Wheeler Alignment (BWA) [26] subsequent variant calling, and was performed by the Core Facility Genomics of the University (Westfälische-Wilhelms Universität Münster) based on the Genome Analysis Toolkit (GATK) best-practice workflow [27] implemented by the Snakemake workflow dna-seq-gatk-variant-calling by Kösters et al. [28, 29].

Further data exploration, calculations and descriptive statistical analyses were performed using Microsoft Excel 2016 and the IBM® SPSS®-Software version 28. A Mann–Whitney *U* test was applied to calculate statistically significant differences between 21 and 120 days in the preliminary study as well as 22 and 92 days in the follow-up study. Statistically significant differences between dry and humid conditions were calculated accordingly.

All analyses were based on read counts at single-nucleotide variant (SNV) sites with regard to GRCh38. Genotype allocations are based on read counts at each position. Genotype 0/0 represents the homozygote allele as given in GRCh38, genotype 0/1 represents the heterozygote genotype with the allele of refGenome38 and the alternative allele, and 1/1 represents the homozygote alternative allele. The total number of reads per SNV position (of all detected alleles) and the number of reads for the alternative allele was used to calculate the percentage of read counts of the variant allele (short: percentage reads of variant, PRV). A PRV of 0% represents the 0/0 genotype, a PRV of 100% represents genotype 1/1, while values in between represent the heterozygous genotype. The day 0 blood sample was considered to show the “true” genotype of the donor. Any differences between day 0 and stored samples were regarded as changes during storage. In addition to changes of the genotype calls between day 0 and stored samples, PRV changes over time were analyzed. Data obtained from whole-genome sequencing contain a plethora of information, which cannot be covered in a single manuscript. This study analyzed single-nucleotide variants (SNV) only while other forms of DNA damage, such as strand breaks, will be the subject of future analyses of the same data set.

Firstly, genotype allocations were analyzed relative to the reference genome. In a second step, SNVs were further characterized regarding affected DNA bases. In a third step, analysis focused on G > A and C > T changes only because such transitions are expected to be the most frequent base substitutions due to hydrolytic attacks. Finally, we identified potential candidate markers for the estimation of TsD based on gradual changes in PRV.

## Results

### Library quality check

#### Preliminary study

Library concentrations varied between 0.48 ng/µl and 0.53 ng/µl. The quality control showed a decrease of average fragment size in the library area between 163 and 951 bp. The fragment size decreased from 457 bp at time point 0 day of storage to 444 bp at time point 21 days of storage to 411 bp at time point 120 days of storage.

#### Follow-up study

Library concentrations varied between 0.36 ng/µl and 8.88 ng/µl. The quality control again showed a decrease of average fragment size in the library area between 200 and 1000 bp. Comparing the median values of the average fragment size of all samples at the different time points, a decrease from 535 bp at time point 0 to 527 bp at time point 1 (22 days) of storage and to 492 bp at time point2 (92 days of storage) was observed.

### Identification of artificial SNVs by WGS

#### Preliminary study

Sequencing of the initial three samples was successful. The sequencing run showed a Q30-value > 94%. Sequencing data were filtered to positions showing differences between the day 0 sample and the two samples stored for 21 and 120 days, respectively. A total of 12,261 base changes between day 0 and day 21 or 120 were identified.

Firstly, genotype allocations were analyzed relative to the reference genome. A first effect of storage was observed by an increase in failed genotype calls (due to no coverage). While all genotypes were called in the day 0 sample, day 21 sample showed 507 and the day 120 sample showed 883 failed genotype callings out of 12,261 variant calls.

While in day 0, the majority of positions showed a homozygote genotype identical to the reference genome GRCh38 (0/0; Table 1), samples stored for 21 or 120 days showed mainly heterozygote genotypes (0/1). Table 1 clearly shows that positions with a 0/0 genotype change into a 0/1 genotype during storage. This effect is visible between day 0 and day 21 while no further increase in 0/1 genotypes was observed between day 21 and day 120. Furthermore, the number of homozygote 1/1 genotypes decreased during storage.

**Table 1:**
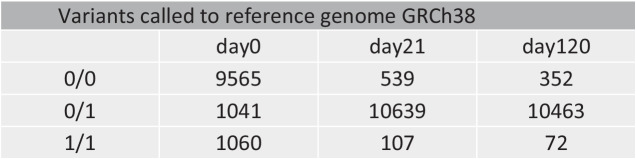
Distribution of genotypes over time — initial study

Since the day 0 blood sample is considered to be the positive control representing the true genotype of the donor, differences between this sample and the stored samples have to be a product of changes during storage time.

We observed the highest number of genotype changes between the day 0 and the day 21 sample. A total of 9270 genotype changed from 0/0 in the day 0 sample to 0/1 in the day 21 sample were observed. Five positions showed a gradual change from genotype 0/0 in the day 0 sample to 0/1 in the day 21 sample to 1/1 in the day 120 sample with a gradually increasing PRV (Table 2). In addition, 989 homozygote 1/1 genotypes in the day 0 sample changed into a 0/1 genotype in the day 21 sample. Of these, 12 positions showed a gradual decrease of PRV and a further change to a 0/0 genotype in the 120 day sample (see Table 2 for details).

**Table 2:**
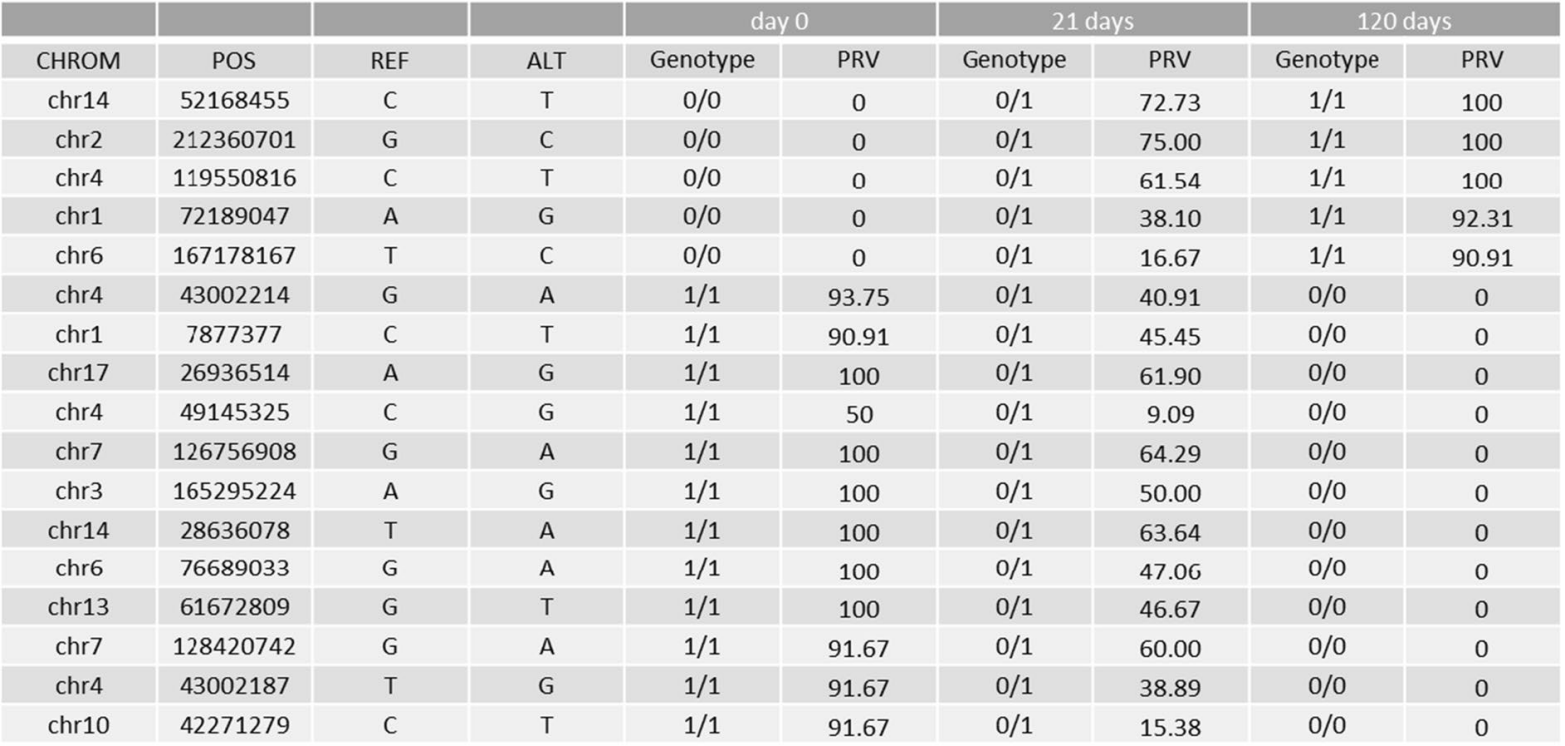
Variants that change from a homozygote genotype in the day 0 sample into the opposite homozygote genotype in the day 120 sample — initial study

**Fig. 1:**
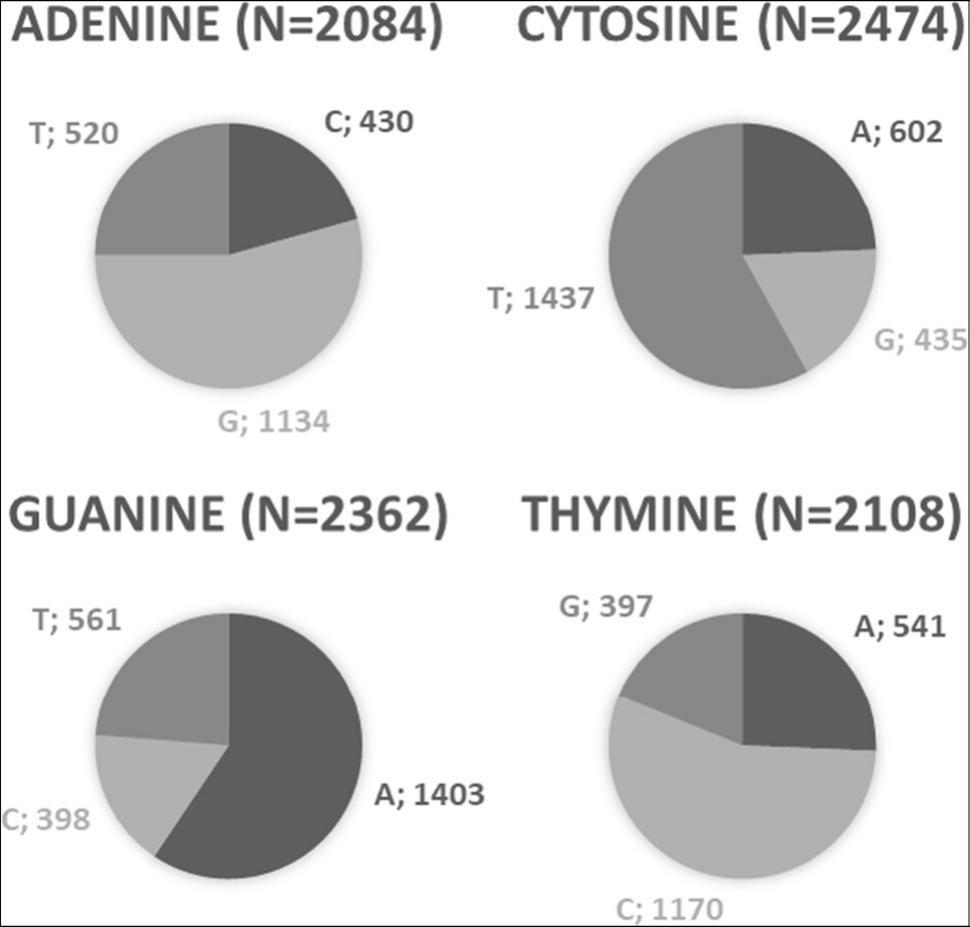
The majority of base changes occur at guanine and cytosine positions comprising mainly G-A and C-T changes. Changes from 0/0 genotypes to 0/1 genotypes between day 0 and day 120

In a second step, SNVs were further characterized. Due to hydrolytic damage of DNA, it was expected to observe mainly G-A and C-T transitions. Analyzing the bases affected by changes induced during storage it was indeed observed that generally more than 50% of changes were G to A and C to T changes or vice versa (Fig. 1):

Figure 1 shows by example bases affected by genotype changes from 0/0 to 0/1 between the day 0 sample and the day 120 sample. All other genotype changes reveal very similar distributions (see Online Resource 1 for details). As expected, changes of guanine and cytosine were found more frequently (*n* = 2474 and *n* = 2362) compared to changes of adenine and thymine (*n* = 2084 and *n* = 2108).

**Fig. 2:**
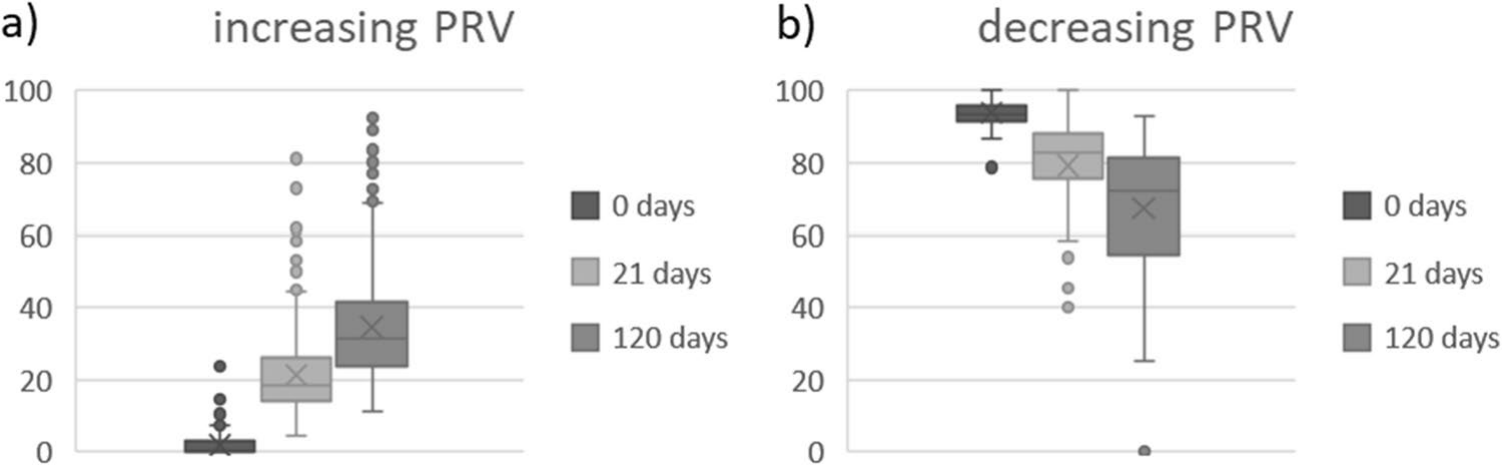
PRV of G > A and C > T changes that show an increase (**a**) or decrease (**b**) over time

In a third step, analysis focused on G > A and C > T changes only, using a threshold of 10 read counts to eliminate changes that can be explained by sequencing errors. Because the majority of changes observed were between the day 0 sample and the day 21 sample and concerned mainly genotype changes from homozygote 0/0 or 1/1 to heterozygote 0/1, these positions are the most interesting for further analysis: a total of 1120 C > T changes and 1134 G > A changes over time were observed in positions showing a 0/0 genotype in the day 0 sample. A total of 108 C > T and 109 G > A changes over time were observed in positions showing a homozygote 1/1 genotype. Of these positions, 605 C > T changes and 627 G > A changes showed an increase or decrease of the relative detection intensity PRV of the new variant (Fig. 2). Statistically significant differences of PRV between the different storage times were observed (ANOVA: *p* < 0.001).

Of all positions depicted in Fig. 2, nine positions were identified that show ideally distributed PRVs over time (Table 3). These positions are even better suited as potential marker candidates for estimation of TsD than positions that show a full genotype change from one homozygote into the opposite homozygote.

**Table 3:**
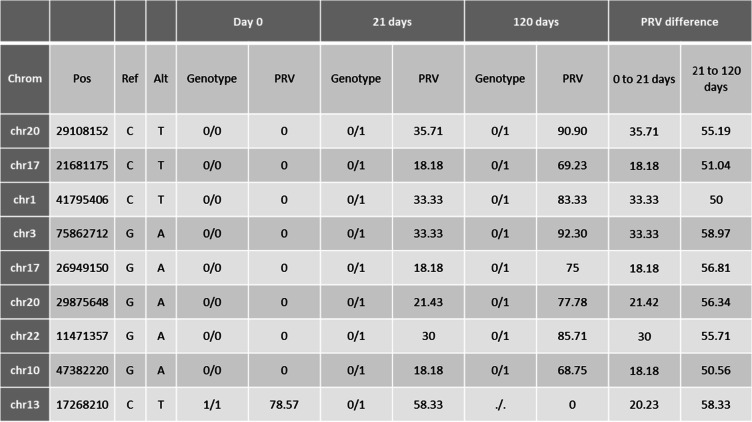
Nine positions that are suitable candidate markers for TsD estimation with gradually changing PRV

Results of this preliminary study confirmed the hypothesis that artificial SNVs do occur during storage of blood samples for up to 120 days and that PRV of such positions increase or decrease over time. It was further confirmed that cytosine and guanine are more susceptible to DNA damage compared to adenine and thymine. Based on these observations, a follow-up study was performed to analyze the effect of different storage conditions (dry and humid) and potential differences between body fluids (blood and saliva).

## Follow-up study

All 10 samples in the follow-up study were successfully sequenced. In this study, Illumina NextSeq 500 System was used to increase coverage of sequencing. Increase in coverage resulted in more SNVs being detected rather than in higher read counts of single positions: a total of 550,448 SNVs found in blood samples and 547,170 SNV in saliva samples that occur in stored samples but not in the fresh samples at day 0. This follow-up study confirmed findings of the preliminary study that there are changes between the day 0 sample (positive control) and stored samples:

Again, a clear increase in failed genotype calls (no coverage) was observed over time (Table 4). The day 0 samples of saliva showed more failed genotyped calls compared to the day 0 sample of blood. The increase of the number of failed genotype calls was stronger under humid conditions compared to dry conditions over the full-storage period.

**Table 4:**
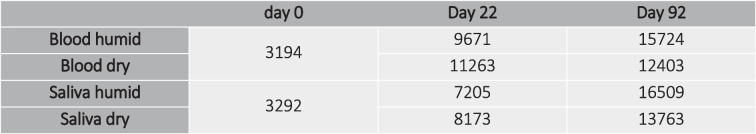
Total number of failed genotype calls (out of 550,448 SNVs in blood and 547,170 SNV in saliva samples) due to no coverage in the follow-up study. An increase of failed genotype calls over time was observed in both samples and in both storage conditions

Again, genotype changes were analyzed first. Similar to the preliminary study, a high number of genotype changes from 0/0 in the day 0 sample towards 0/1 genotypes in the day 22 samples (both humid and dry) was observed. The strongest increase, however, was observed regarding the number of 1/1 genotypes in blood samples between the day 0 sample and the day 22 samples (both humid and dry). This effect was not visible in saliva samples (Table 5). This might be due to a surprisingly higher number of 1/1 genotypes in the day 0 sample of saliva than in the day 0 sample of blood (numbers highlighted in bold). Similar to genotype changes described in the preliminary study, the majority of changes was detected within the first 22 days of storage. Interestingly, there were no clear changes in the total number of genotypes over time in saliva samples. Furthermore, a significantly lower number of changes from 0/1 genotypes to 1/1 genotypes were observed in saliva samples compared to blood samples, after both 22 and 92 days of storage.

**Table 5:**
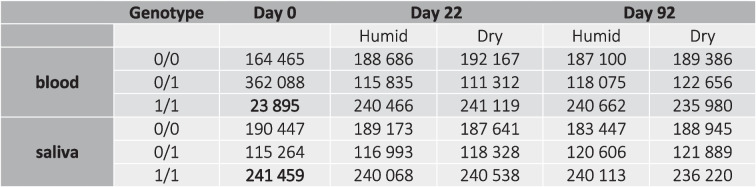
Distribution of genotypes over time — follow-up study

Extreme genotype changes from a homozygote genotype in the day 0 sample to a 0/1 genotype in the day 22 sample and the opposite homozygote in the day 92 sample were observed. Table 6 gives an overview of the total number of positions detected, while full details of these positions are given in the Online Resource 2. Most positions identified in the dry and humid samples were not identical. In blood, only two identical positions under humid and dry conditions were identified; both changed their genotype from 0/0 to 1/1. In saliva, there were three matching sites between conditions changing their genotype from 1/1 to 0/0.

**Table 6:**
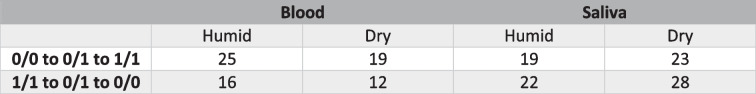
Total number of positions that change from a homozygote genotype in the day 0 sample into the opposite homozygote genotype in the day 92 sample — follow-up study

**Fig. 3:**
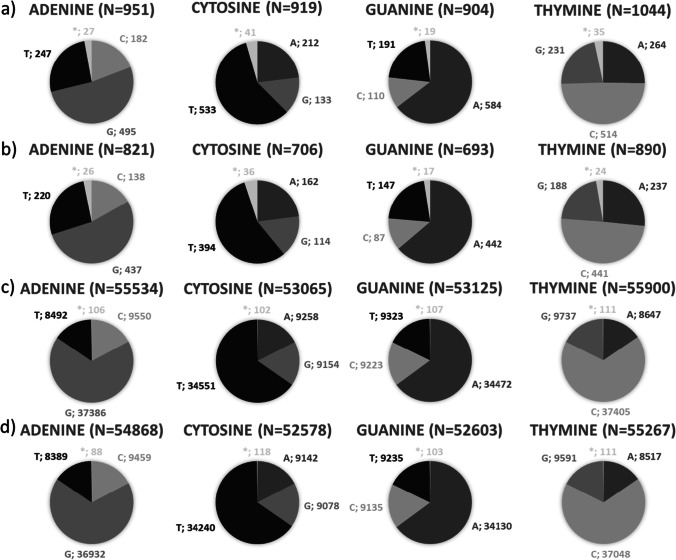
Genotype changes from day 0 = 0/1 to day 92 = 1/1 in the follow-up study. (**a**) Saliva humid (total *n* = 3818), (**b**) saliva dry (total *n* = 3110), (**c**) blood humid (total *n* = 217,624), and (**d**) blood dry (total *n* = 215,316). Majority of changes represent G-A or C-T changes

Analyzing the bases affected by changes over time, it was again observed that more than 50% of changes were G > A or C > T changes in both saliva and blood samples. This was true for changes between day 0 and day 22 as well as day 0 and day 92 and between day 22 and day 92, respectively. Figure 3 shows by example the variations between the positive control and the day 92 samples. Finally, a significantly stronger increase in the relative number of spanning deletions designated as asterisk (*) over time was observed in saliva samples compared to blood samples (chi^2^ test, *p* < 0.05). In humid samples, we observed only 3818 changes between day 0 and day 92 in saliva, while in blood, 217,624 changes were observed (see Fig. 3). A similar effect was observed for dry samples (3110 changes in saliva versus 215,316 changes in blood); see Online Resource 3 for further details.

**Fig. 4:**
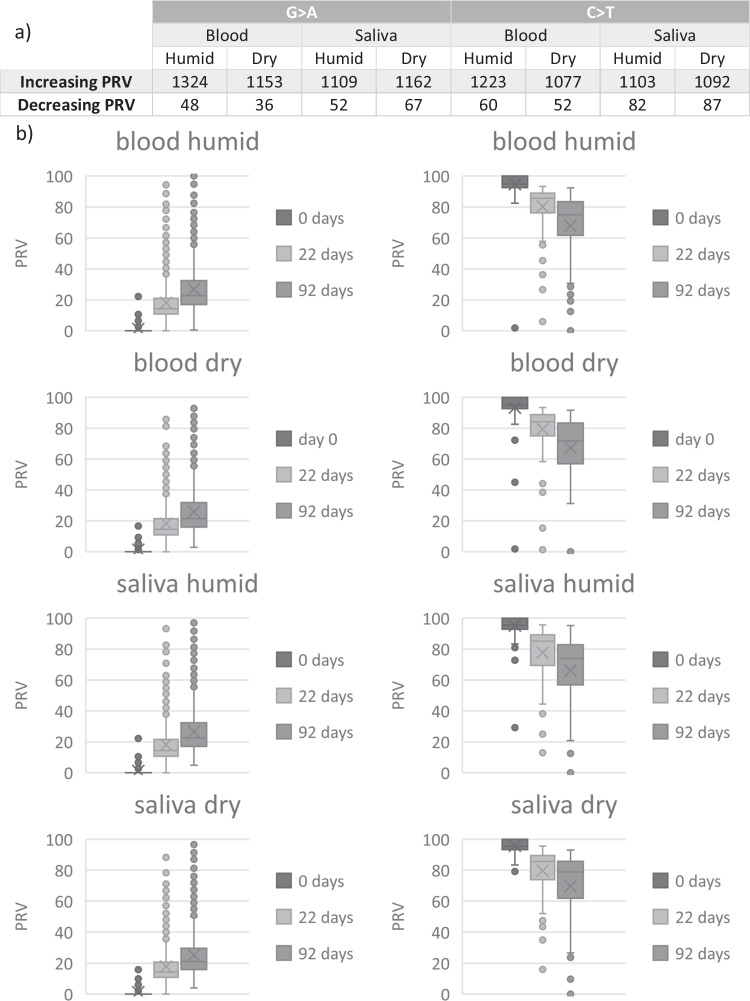
(**a**) Total numbers of C > T and G > A changes that exceed a threshold of 10 read counts and show an increase or decrease of PRV over time, separated by sample origin and storage condition. (**b**) PRV of C > T and G > A changes that show an increase or decrease over time for each combination of biological matrix and storage condition.

In a further step, analysis focused again on G > A and C > T changes only, using a threshold of 10 read counts to eliminate changes that can be explained by sequencing errors. Because the majority of changes observed were between the day 0 sample and the day 22 sample, similar to the preliminary study, these positions remain the most interesting for further analysis. In accordance with the preliminary study, we analyzed increased and decreased PRV, in positions showing a homozygote genotype in the day 0 samples. In each combination of biological matrix and storage condition, over 1000 G > A and C > T changes were identified that show an increase or decrease in PRV over time (ANOVA: *p* < 0.001 for every combination; Fig. 4).

Similar to the preliminary study, we further analyzed all positions detailed in Fig. 4 above to identify positions that show a decrease or increase in PRV that is ideally distributed PRVs over time. These positions are better suited as potential marker candidates for the estimation of longer TsD while positions that show a full genotype change from one homozygote into the opposite homozygote might be more suitable for shorter TsD.

Numerous such positions were identified, which are highly promising candidate markers for TsD estimation. Details on these positions are given as Online Resource 4.The main aim of this follow-up study, however, was to identify potential markers for the estimation of TsD. Ideally, such a marker is suitable for blood and saliva and is not affected by storage conditions. A total of 11 SNVs were identified that fulfill these conditions (Table 7). Interestingly, all these SNVs represent G > A or C > T changes and comprise positions with increasing PRV only. These positions are considered to be potential candidate markers for future assays to determine the age of a blood or saliva trace.

**Table 7:**
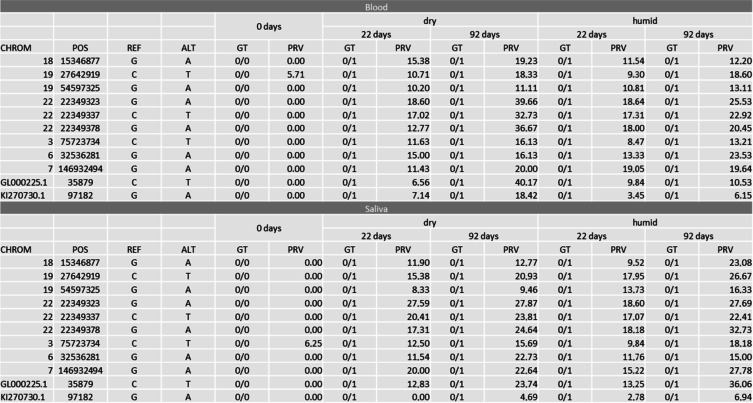
Potential markers for future TsD assays identified in the follow-up study. Positions displayed show an increase in PRV over time in both saliva and blood samples, irrespective of the storage condition

**Fig. 5:**
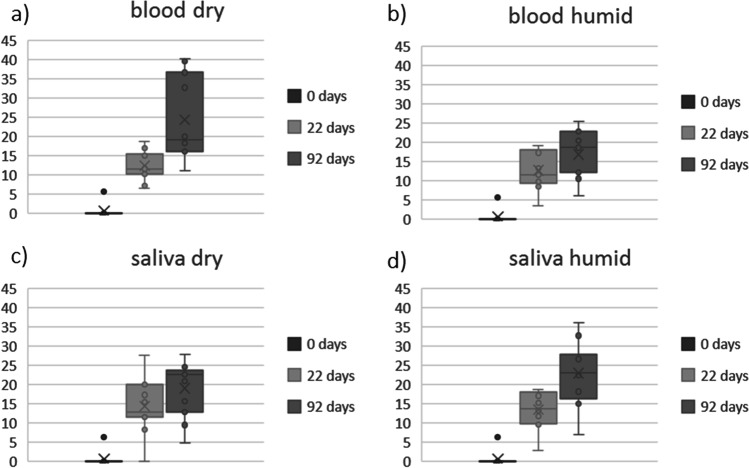
Boxplots of PRV changes of all positions displayed in Table 7

Further analysis of these most promising positions for TsD estimation revealed that all of them comprise clear increasing PRV over time (Fig. 5) with statistically significant changes in every combination (*p* < 0.001).

## Discussion

### Library quality check

The decrease in fragment size that was observed in the library quality check of the preliminary study was confirmed by the results of the follow-up study. While no statistical calculation of significance was performed due to the small sample size, the results confirm the expectation that samples stored for longer durations seem to be more sensitive for mechanical shearing because of the formation of abasic sites caused by hydrolytic attack of DNA. Hianik et al. [30] showed that abasic sites lead to a dramatic destabilization of DNA. Consequently, there are shorter library fragments produced by library preparation in samples that were stored for longer time periods.

### Identification of artificial SNVs by WGS

#### Total number of artificial SNVs

Due to an increased coverage in the follow-up study, a higher number of artificial SNVs were identified compared to the preliminary study. Differences in the total number of SNVs detected in the day 0 blood samples of the preliminary study and the day 0 blood sample of the follow-up study were observed, even though the samples originate from the same donor and the day 0 blood sample was considered to reflect the true genotype of the donor. The data analysis pipeline leads to such differences between the two studies: only positions that show a difference between the three time points analyzed were called. Consequently, positions listed after filtering of all sequencing data for the day 0 blood sample do not reflect all positions in which the donor carries a different allele than the GRCh38, but all positions in which the day 0 sample differs from either or both stored samples. Thus, the differences observed between the two studies were expected.

In general, it is not possible to determine the amount of “artificial SNVs” that actually originate from sequencing errors rather than from real sequence changes as a result of DNA damage. Stoler et al. recently discussed the challenges of determining an exact error rate for sequencing runs but found that the NovaSeq6000 sequencer used for our follow-up study showed a fourfold lower median error rate compared to the NextSeq500 used in our preliminary study [31]. Even though we applied a threshold of ten reads [32] to eliminate some stochastic effects, we expect a slightly higher number of falsely called artificial SNVs in our preliminary study compared to our follow-up study due to the different sequencers. All sequencing in the preliminary as well as the follow-up study were conducted in single sequencing runs. Consequently, we expect comparable error rates for all samples within each study. The analysis pipeline focused on sequence differences between the time points and differences found between these time points cannot be explained by sequencing error rates but were considered to be time-dependent.

In both the preliminary and follow-up studies, the majority of genotype changes over time happened between day 0 and day 21. A surprising difference was observed between the day 0 samples of blood and saliva, with fewer 1/1 genotypes in blood compared to saliva (Table 5). After a storage period of 22 days, both blood samples (humid and dry) showed an increase of 1/1 genotypes equal to the number of 1/1 genotypes in all three saliva samples. Interestingly, saliva samples did not show any further genotype changes over time. While it is well known that the majority of differences between samples from the same donor can be explained by differences in filtering data in the initial data analysis pipeline after WGS, this cannot explain the differences observed between over time in this study: our follow-up study used an identical data analysis pipeline for blood and saliva samples, which were analyzed in the same sequencing run. The day 0 sample was expected to show no signs of DNA degradation, particularly not though hydrolysis. Nevertheless, a saliva sample, or more precisely in this case a mouth mucosa sample collected by rubbing the inside of the donor’s cheek, might already have been altered by hydrolysis. Even though scraping the inside of the inner cheek reaches mainly the non-keratinized squamous epithelium with intact cells, there will most certainly always be a proportion of liquid saliva present, carrying cell-free DNA from keratinized cells from other areas of the oral cavity. We hypothesize that DNA might already have suffered from hydrolytic attacks within the mouth and might already show base changes. Consequently, this means that while the day 0 sample of blood truly represents the donor’s genotype and comprise mainly intact, living cells at the time point of collection, the “day 0” saliva sample is not really a time point 0 sample, but might contain extracellular DNA that is stored under humid conditions for at least several hours. Further studies on sequencing differences between saliva and blood samples are needed to prove this point. Samson et al. [33] demonstrated that short sequences of non-human origin can align to the human reference genome and therefore result in false-positive variant and genotype calling. They and other studies like Trost et al. [34] concluded that analyzing DNA from blood samples is the best option for variant analysis in WGS.

#### Increase of failed genotype calls over time

Both studies showed an increase of failed genotype calls (no sequencing coverage) over time in all biological matrices and storage conditions. Contrary to microdeletions, these positions show no sequencing coverage. One explanation might be that base changes increasingly affect positions within primer binding regions, thus inhibiting the amplification and sequencing reaction. Furthermore, depurination leads to DNA fragmentation and this obviously leads to decreased amplification efficiency of longer amplicons during library preparation.

In the follow-up study, a much higher number of failed genotype calls were observed in the day 0 saliva sample compared to the day 0 blood sample. Again, this might be due to beginning degradation in the saliva/mouth mucosa sample as discussed above.

#### Genotype changes during storage

In both studies, genotype changes over time were observed. The most extreme changes comprise gradual homozygote switches, i.e., from a homozygote 0/0 or 1/1 genotype into a 0/1 genotype and further on into the opposite homozygote genotype after the full length of storage. These positions show a very clear storage effect and might be considered the most suitable candidate markers for future TsD estimation. Furthermore, the majority of genotype changes in both studies was observed between the day 0 samples and the day 21 or day 22 samples, respectively. Changes observed here, however, are fast and a complete change was reached during the storage time analyzed here. Thus, from data obtained here it remains unclear how fast exactly this change occurs. Consequently, such positions might be useful candidate markers in the estimation of very short TsD periods and further analyses of such positions with more time points, particularly within the first 3 weeks of storage, will shed light on the suitability of these positions as TsD markers.

For longer storage periods, however, the ideal candidate markers for TsD do not necessarily show a complete genotype change, but a slow and gradual change of PRV over time (see below).

#### Analysis of base changes over time

The majority of artificial SNVs comprised G to A and C to T changes, respectively, and vice versa. This is in line with the well-accepted concept that G-A and C-T transition mutations are generated at higher frequency transversion mutations, due to their specific molecular mechanisms. Earlier studies provided by, e.g., Rathburn et al., Binladen et al., and Brotherton et al. [32, 35, 36], also pointed out that depurination by hydrolytic attacks more likely deplete guanine and deamination leads to cytosine to thymine variants.

Our results obtained from both blood and saliva samples sequenced with a higher coverage and a more sensitive instrument as part of the follow-up study confirmed the assumption that C and G are the bases effected with the highest frequency. Consequently, such storage-induced artificial SNVs might be the most promising candidate markers for TsD estimation. Further analysis of our dataset focused mainly on positions showing such transitions. Rathburn et al. [32] observed a higher number of type 1 transitions (A > G and T > C) during storage compared to type 2 transition (G > A and C > T). Our data, however, confirm data presented by Hofreiter et al. [37] with observation of a higher number of type 2 transitions. Rathburn et al. [32] explain such differences by different sequencing technologies and platforms.

#### Potential candidate markers for TsD estimation

In the preliminary study, nine positions were identified that showed gradual changes of PRV over time making them potential candidates for TsD estimation in blood samples. The sequencing coverage and sensitivity was slightly lower in the preliminary study and further analyses in a larger sample set and with increased sequencing coverage are needed to confirm their potential use as TsD markers (see below). The follow-up study identified markers that might enable estimation of TsD in two different body fluids (blood and saliva) and two different storage conditions (humid and dry).

As mentioned above, there might be a difference between positions which are suitable for estimating short-term TsD and the ones more suitable for longer term TsD periods. While fast and complete genotype changes as described above might be more suitable for estimating short-term TsD, the aim of the follow-up study was to identify candidate markers for medium- to long-term TsD which are suitable for both saliva and blood samples. The introduction of a threshold of 10 read counts eliminated artificial SNVs that cannot be distinguished from mere sequencing errors, as described by Rathburn et al. [32].

We identified 11 positions which fulfill such criteria and show an ideal distribution of PRV changes over time. In this case, ideal means that in positions with a 0/0 genotype in the day 0 sample, the PRV of the alternative allele (1) increases relative to the storage time, e.g., to up to 20% in the day 22 sample to close to 100% in the day 92 sample. These 11 positions showed such gradually decreasing or increasing PRV over time irrespective of the biological matrix and the storage conditions. The positions describe the evolution of homozygous-heterozygous only. These positions can be informative for TsD estimation even if the respective position in an original trace DNA profile is heterozygote because gradual changes in PRV can still be observed and are more informative than genotype changes, as mentioned above. We are aware that focusing on homozygote positions introduces an amount of bias into the analyses and further positions with gradual PRV changes over time might be found in future analyses of the same data set.

Future analysis of these 11 markers in a large sample set comprising saliva and blood samples from numerous donors is necessary to assess their true potential as candidate markers for an assay to estimate TsD in biological traces.

## Conclusion

The aim of this study was to identify artificial single-nucleotide variants (SNVs) that are products of DNA damage over time. Future analyses of the same data set can focus on the analysis of indels and strand breaks, for example. In this study, artificial SNVs were observed in high numbers, mainly affecting guanine and cytosine, as expected. A stepwise search revealed potential candidate markers for estimating short-term TsD and medium- to long-term TsD. Further investigation of these candidate markers in targeted sequencing approaches including multiple donors will reveal their suitability for TsD estimation. After confirmation of the candidate markers and development of a prediction algorithm, full forensic validation is needed to understand the influence of parameters such as various storage conditions, individual characteristics of the donor (e.g., sex and age), and different biological matrices.

## Supplementary Information

Below is the link to the electronic supplementary material.

Supplementary file1 (PDF 596 KB)

Supplementary file2 (PDF 542 KB)

Supplementary file3 (PDF 1756 KB)

Supplementary file4 (PDF 750 KB)

## Data Availability

The datasets generated during and/or analyzed during the current study are available from the corresponding author on reasonable request.
