# Supplementary material for: Whole-genome sequencing of artificial single-nucleotide variants induced by DNA degradation in biological crime scene traces
Source: Int J Legal Med. 2022 Nov 10;137(1):33–45. doi: 10.1007/s00414-022-02911-0 (PMC9816238; doi:10.1007/s00414-022-02911-0)
Supplement: Supplementary file 1 — Supplementary file1 (PDF 596 KB) [file 414_2022_2911_MOESM1_ESM.pdf]

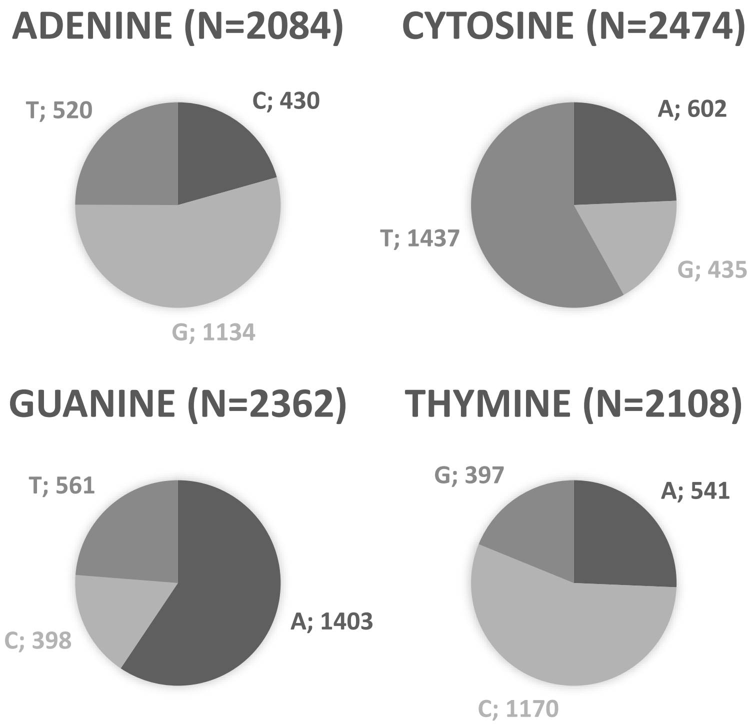

day0=0/0; day120=0/1

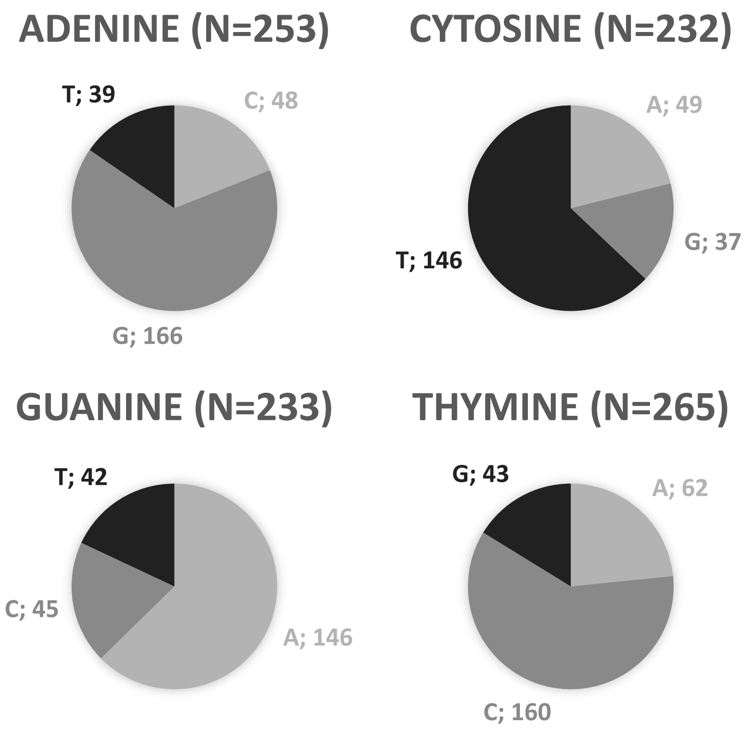

day0=1/1; day21=0/1

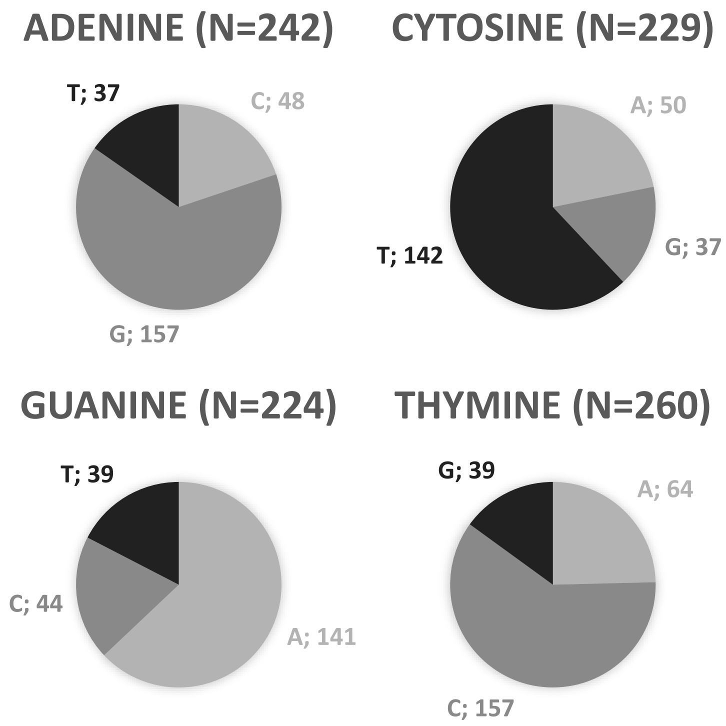

<u>day0=1/1; day120=0/1</u>

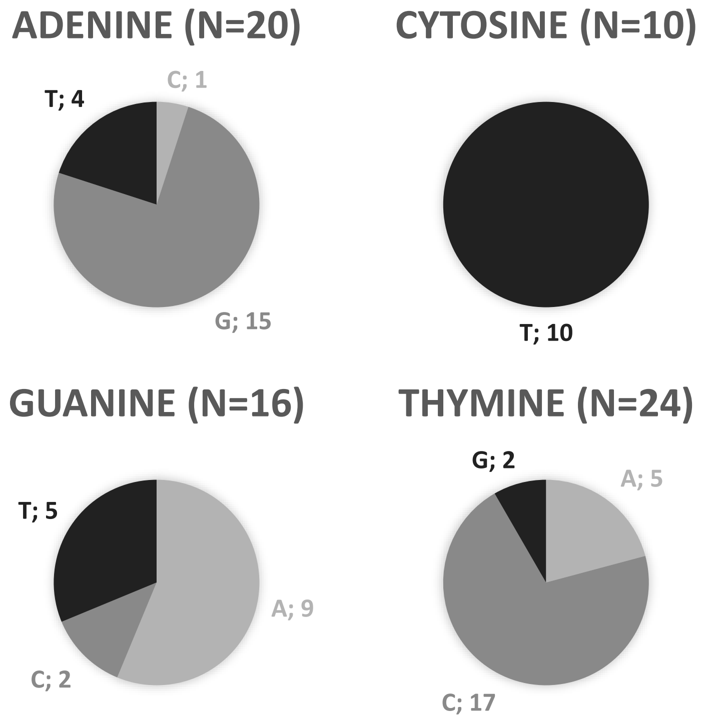

<u>day0=0/1; day21=1/1</u>

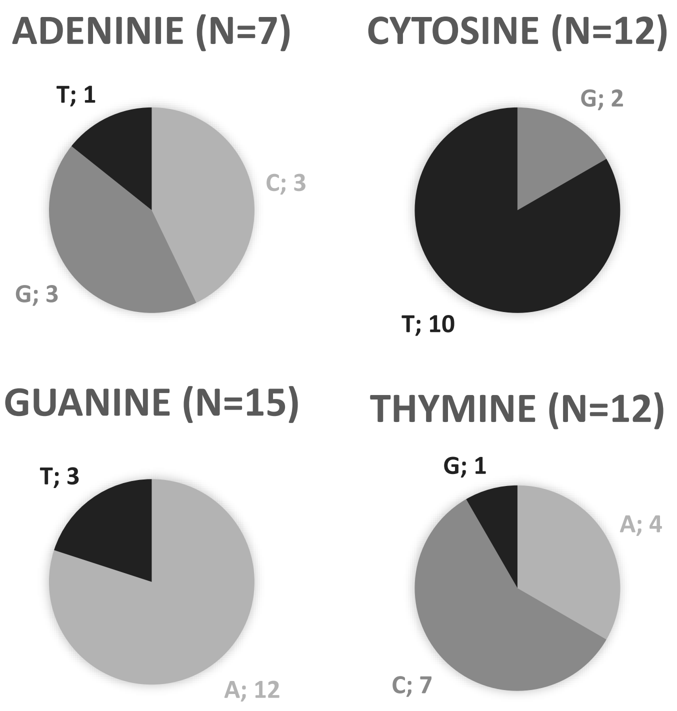

day0=0/1; day120=1/1

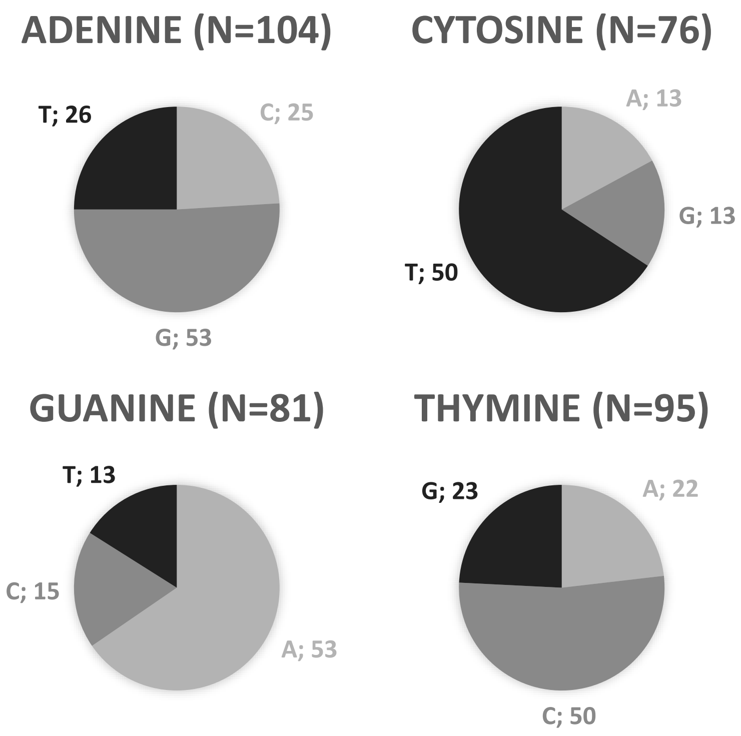

day0=0/1; day21=0/0

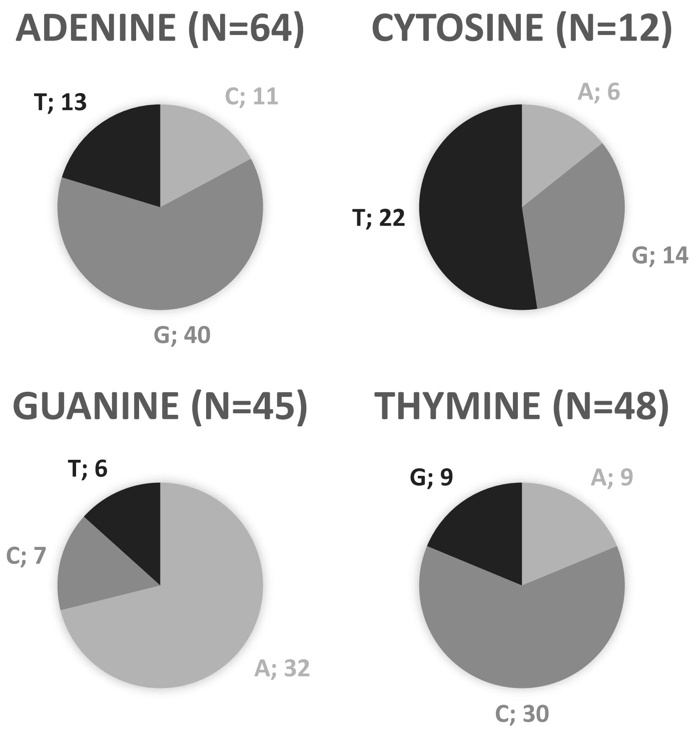

day0=0/1; day120=0/0

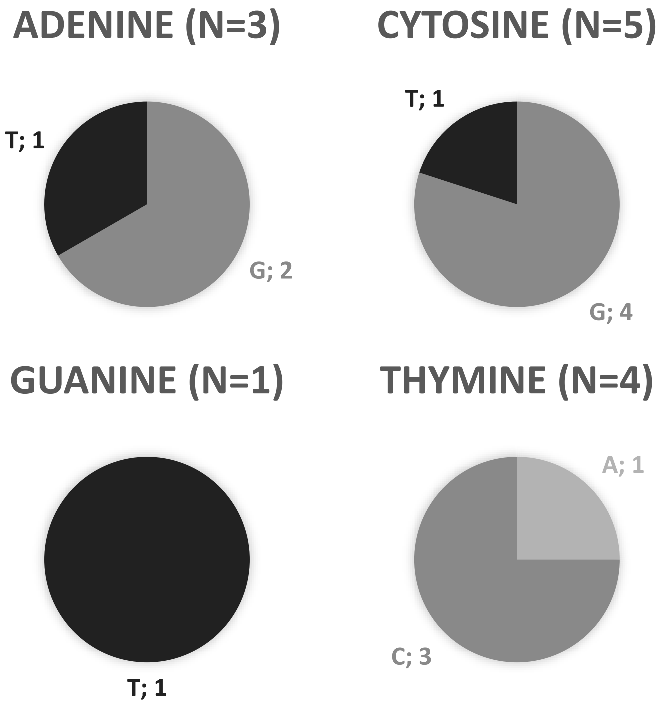

day0=1/1; day21=0/0

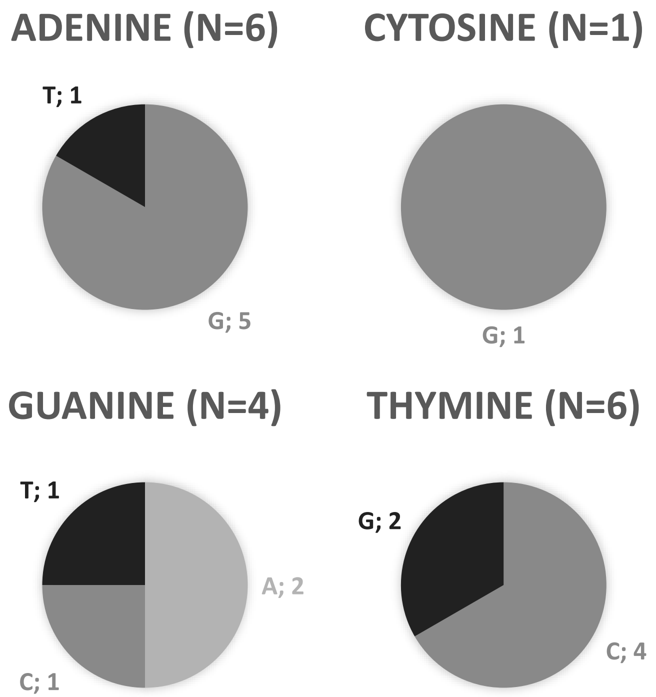

day0=1/1; day120=0/0

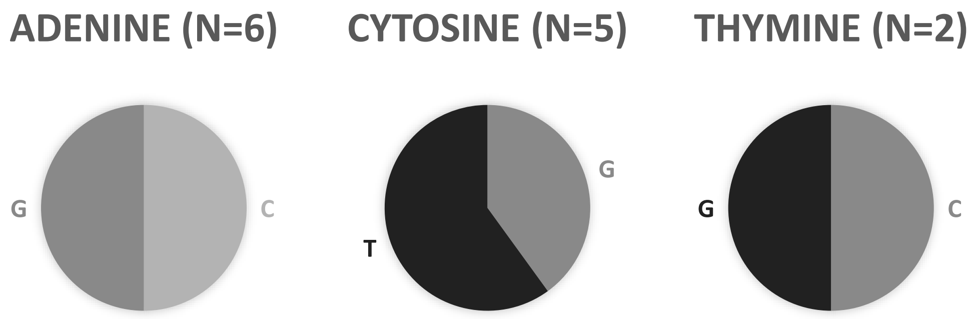

day0=0/0; day21=1/1 (no variants in guanine)

**A>G**	2
**C>T**	2
**G>C**	1
**T>C**	1

day0=0/0; day120=1/1
