# Supplementary material for: Whole-genome sequencing of artificial single-nucleotide variants induced by DNA degradation in biological crime scene traces
Source: Int J Legal Med. 2022 Nov 10;137(1):33–45. doi: 10.1007/s00414-022-02911-0 (PMC9816238; doi:10.1007/s00414-022-02911-0)
Supplement: Supplementary file 2 — Supplementary file2 (PDF 542 KB) [file 414_2022_2911_MOESM2_ESM.pdf]

blood humid										
CHROM	POS	REF	ALT	QUAL	GT.156	PRV 0 days	GT.152	PRV 22 days	GT.148	PRV 92 days
1	39914524	T	G	2119.84	0/0	0,00	0/1	81,25	1/1	81,25
10	71759852	C	T	3353.87	0/0	0,00	0/1	78,57	1/1	78,57
11	1095960	T	C	1783.74	0/0	0,00	0/1	47,62	1/1	47,62
12	61526033	G	A	972.78	0/0	0,00	0/1	38,10	1/1	38,10
13	49805119	G	A	2399.87	0/0	0,00	0/1	47,83	1/1	47,83
15	91048340	T	C	1924.16	0/0	0,00	0/1	28,57	1/1	28,57
16	27146900	T	C	1257.78	0/0	13,64	0/1	30,77	1/1	30,77
17	82151215	A	G	5680.17	0/0	0,00	0/1	66,67	1/1	66,67
17	82151217	C	A	6056.82	0/0	0,00	0/1	75,00	1/1	75,00
2	227497739	T	G	3622.97	0/0	0,00	0/1	73,33	1/1	73,33
2	230466204	C	A	1041.28	0/0	0,00	0/1	24,14	1/1	24,14
21	30816284	A	T	3050.98	0/0	0,00	0/1	50,00	1/1	50,00
4	49121061	T	A	131241.0	0/0	7,95	0/1	29,63	1/1	29,63
4	83299368	A	G	4348.23	0/0	0,00	0/1	84,62	1/1	84,62
4	187828524	A	G	3564.37	0/0	0,00	0/1	84,62	1/1	84,62
4	190065458	C	T	2239.15	0/0	0,00	0/1	84,21	1/1	84,21
4	190065475	G	A	3027.14	0/0	0,00	0/1	83,33	1/1	83,33
4	190065481	C	T	2980.69	0/0	0,00	0/1	83,33	1/1	83,33
4	190065492	C	G	2983.69	0/0	0,00	0/1	83,33	1/1	83,33
4	190065502	G	A	2847.86	0/0	0,00	0/1	77,78	1/1	77,78
4	190065503	C	A	2847.86	0/0	0,00	0/1	77,78	1/1	77,78
5	22698743	T	A	1672.68	0/0	0,00	0/1	83,33	1/1	83,33
6	115299630	G	A	3241.34	0/0	0,00	0/1	69,23	1/1	69,23
6	148324137	C	A	1920.05	0/0	0,00	0/1	54,55	1/1	54,55
7	158407676	G	C	2186.39	0/0	0,00	0/1	47,37	1/1	47,37
11	22986487	T	C	3282.34	1/1	100,00	0/1	55,56	0/0	0,00
11	42580062	A	C	1564.19	1/1	88,89	0/1	50,00	0/0	0,00

15	54172705	T	C	5671.2	1/1	100,00	0/1	75,00	0/0	0,00
15	54172707	T	G	5671.2	1/1	100,00	0/1	75,00	0/0	0,00
20	63445759	G	A	6507.09	1/1	94,12	0/1	86,36	0/0	0,00
20	63445764	A	G	6231.61	1/1	93,75	0/1	84,09	0/0	0,00
20	63445765	C	T	6273.05	1/1	93,55	0/1	84,09	0/0	0,00
20	63445766	C	T	6153.64	1/1	93,33	0/1	84,09	0/0	0,00
20	63445769	G	A	6186.73	1/1	93,55	0/1	84,09	0/0	0,00
5	49666291	C	A	18314.8	1/1	95,24	0/1	52,88	0/0	0,00
7	152796876	T	G	2186.95	1/1	91,67	0/1	30,77	0/0	0,00
8	34102150	T	G	2655.25	1/1	90,48	0/1	72,00	0/0	0,00
8	88639146	T	A	280.26	1/1	57,14	0/1	54,55	0/0	16,00
X	29803366	T	C	4797.66	1/1	100,00	0/1	64,71	0/0	0,00
X	66337760	G	A	2590.18	1/1	100,00	0/1	54,55	0/0	0,00
KI270438.1	106965	G	C	631479.0	1/1	94,45	0/1	85,93	0/0	0,00
blood dry										
CHROM	POS	REF	ALT	QUAL	GT.156	PRV 0 days	GT.154	PRV 22 days	GT.150	PRV 92 days
X	103483997	A	T	859.41	0/0	15,38	0/1	26,67	1/1	100,00
13	22762661	T	G	4768.07	0/0	0,00	0/1	42,86	1/1	100,00
22	43165913	A	G	4401.57	0/0	0,00	0/1	43,75	1/1	58,54
4	83299368	A	G	4348.23	0/0	0,00	0/1	38,46	1/1	95,24
6	6603611	A	C	3882.07	0/0	0,00	0/1	42,86	1/1	100,00
19	410809	A	G	3755.02	0/0	0,00	0/1	88,89	1/1	100,00
2	227497739	T	G	3622.97	0/0	0,00	0/1	80,00	1/1	100,00
6	6603606	A	C	3485.07	0/0	0,00	0/1	35,71	1/1	100,00
8	142975055	G	A	3252.93	0/0	0,00	0/1	61,29	1/1	96,15
8	142975048	G	A	3118.37	0/0	0,00	0/1	69,70	1/1	92,86
4	183350055	T	C	2800.05	0/0	0,00	0/1	64,71	1/1	100,00
6	23125380	A	C	2577.55	0/0	0,00	0/1	62,50	1/1	86,96
6	120912516	G	A	2571.35	0/0	0,00	0/1	38,10	1/1	88,24

11	21745052	T	G	2164.25	0/0	0,00	0/1	61,54	1/1	86,21
3	150493153	T	C	1915.99	0/0	0,00	0/1	27,78	1/1	92,86
19	3332267	C	T	1773.96	0/0	0,00	0/1	25,00	1/1	90,91
15	24835000	G	C	1483.96	0/0	0,00	0/1	53,85	1/1	100,00
21	45746193	C	T	1238.11	0/0	0,00	0/1	68,42	1/1	91,67
4	1531316	T	G	1176.68	0/0	0,00	0/1	11,54	1/1	100,00
1	42385827	C	T	5064.9	1/1	100,00	0/1	76,47	0/0	0,00
15	100001992	C	A	2163.77	1/1	100,00	0/1	72,73	0/0	21,05
2	89671813	G	A	7801.27	1/1	100,00	0/1	36,36	0/0	0,00
21	42994322	G	A	1906.61	1/1	100,00	0/1	61,54	0/0	0,00
21	42994323	T	G	1906.62	1/1	100,00	0/1	61,54	0/0	0,00
21	43336855	C	T	6368.4	1/1	100,00	0/1	81,82	0/0	0,00
4	115022374	T	C	3705.17	1/1	100,00	0/1	40,54	0/0	0,00
7	47389118	A	C	2961.92	1/1	100,00	0/1	66,67	0/0	0,00
7	47389119	A	G	2924.31	1/1	100,00	0/1	69,23	0/0	0,00
8	7381029	A	C	2464.01	1/1	88,89	0/1	61,54	0/0	0,00
X	103483994	C	T	3087.0	1/1	90,91	0/1	72,73	0/0	0,00
KI270442.1	16899	A	G	646.52	1/1	100,00	0/1	15,38	0/0	0,00
saliva humid										
CHROM	POS	REF	ALT	QUAL	GT.157	PRV 0 days	GT.153	PRV 22 days	GT.149	PRV 92 Days
12	12801903	T	*	416.98	0/0	0,00	0/1	29,41	1/1	100,00
12	69362984	T	A	2695.63	0/0	0,00	0/1	30,00	1/1	100,00
14	103302533	T	C	1791.55	0/0	0,00	0/1	41,38	1/1	94,74
15	20342259	A	G	6850.85	0/0	0,00	0/1	79,12	1/1	95,16
16	88251556	T	G	2307.42	0/0	0,00	0/1	89,47	1/1	94,74
18	12211828	T	C	1151.29	0/0	0,00	0/1	21,05	1/1	100,00
19	15616116	C	T	1212.49	0/0	0,00	0/1	46,67	1/1	91,67
2	3199831	G	T	5030.67	0/0	0,00	0/1	83,33	1/1	94,74
2	43308758	C	A	2432.11	0/0	0,00	0/1	45,00	1/1	100,00

2	227497740	T	G	561.97	0/0	0,00	0/1	22,22	1/1	100,00
4	181605532	A	T	2747.23	0/0	0,00	0/1	62,50	1/1	100,00
5	100378909	A	C	1383.04	0/0	0,00	0/1	40,00	1/1	89,47
6	83027462	G	A	2638.96	0/0	0,00	0/1	83,33	1/1	100,00
6	106130787	T	C	1408.34	0/0	0,00	0/1	17,86	1/1	88,24
7	2491839	T	C	1135.87	0/0	0,00	0/1	41,67	1/1	100,00
8	7381029	A	C	2464.01	0/0	0,00	0/1	38,89	1/1	93,33
8	47956978	C	A	2536.44	0/0	0,00	0/1	29,41	1/1	100,00
Y	11292489	C	T	9894.13	0/0	0,00	0/1	6,50	1/1	100,00
Y	56829566	C	T	15393.5	0/0	0,00	0/1	12,63	1/1	100,00
1	1981466	C	A	4345.33	1/1	100,00	0/1	77,42	0/0	0,00
1	1981476	T	A	3764.87	1/1	100,00	0/1	77,27	0/0	0,00
1	1981479	C	G	3342.87	1/1	100,00	0/1	73,68	0/0	0,00
1	1981482	G	A	2907.87	1/1	100,00	0/1	70,59	0/0	0,00
1	112219219	A	C	438.37	1/1	88,89	0/1	16,67	0/0	0,00
11	17711478	C	T	819.64	1/1	90,91	0/1	44,44	0/0	0,00
12	130392780	A	G	2297.91	1/1	91,67	0/1	50,00	0/0	0,00
14	103302534	T	C	634.4	1/1	92,86	0/1	25,00	0/0	0,00
14	105703230	T	A	953.4	1/1	90,00	0/1	40,00	0/0	0,00
17	3375247	C	T	1033.53	1/1	80,00	0/1	41,18	0/0	0,00
17	47359518	T	G	2823.71	1/1	91,67	0/1	41,94	0/0	13,33
17	53981464	T	G	3053.71	1/1	91,30	0/1	75,00	0/0	0,00
2	43308757	C	A	1967.34	1/1	100,00	0/1	55,00	0/0	0,00
2	47085051	A	T	1714.22	1/1	100,00	0/1	54,84	0/0	0,00
20	37603410	T	C	3359.04	1/1	100,00	0/1	46,67	0/0	0,00
4	49645539	G	C	3311.73	1/1	46,15	0/1	80,95	0/0	0,00
4	131962788	A	G	2815.02	1/1	100,00	0/1	75,00	0/0	0,00
4	179037032	T	C	2901.75	1/1	96,88	0/1	85,71	0/0	0,00
4	181605536	A	T	3567.26	1/1	100,00	0/1	31,25	0/0	0,00

6	31338358	T	C	3320.13	1/1	100,00	0/1	12,90	0/0	0,00
7	91013658	T	G	4029.47	1/1	96,15	0/1	78,13	0/0	0,00
8	124865502	T	C	1718.07	1/1	92,31	0/1	66,67	0/0	0,00
saliva dry										
CHROM	POS	REF	ALT	QUAL	GT.157	PRV 0 days	GT.155	PRV 22 days	GT.151	PRV 92 days
10	38528054	C	G	83473.5	0/0	0,00	0/1	82,46	1/1	90,00
10	38528169	A	T	111384.0	0/0	0,00	0/1	83,45	1/1	89,68
10	98852115	G	A	3311.02	0/0	0,00	0/1	37,50	1/1	100,00
11	120328869	A	T	3219.28	0/0	0,00	0/1	72,22	1/1	94,44
11	121765655	C	*	189.01	0/0	0,00	0/1	52,63	1/1	75,00
12	16215231	C	A	1003.51	0/0	0,00	0/1	82,35	1/1	100,00
14	89511276	T	C	3000.96	0/0	0,00	0/1	61,11	1/1	100,00
14	89511280	T	C	2843.4	0/0	0,00	0/1	55,56	1/1	100,00
14	89511284	T	C	2864.91	0/0	0,00	0/1	50,00	1/1	100,00
16	138607	G	T	5572.61	0/0	0,00	0/1	44,12	1/1	93,33
18	61538582	T	G	3093.88	0/0	0,00	0/1	61,90	1/1	100,00
18	63086836	C	T	3122.21	0/0	0,00	0/1	20,83	1/1	75,00
19	410759	G	A	6189.16	0/0	0,00	0/1	78,26	1/1	96,55
19	410796	C	A	4476.2	0/0	0,00	0/1	77,78	1/1	100,00
2	76366234	C	T	2149.09	0/0	0,00	0/1	88,24	1/1	100,00
20	3441132	G	A	3413.61	0/0	0,00	0/1	46,34	1/1	100,00
5	174743308	T	C	1955.7	0/0	0,00	0/1	84,21	1/1	92,31
6	114423468	T	G	1238.85	0/0	0,00	0/1	34,78	1/1	85,19
7	24239714	T	A	1900.1	0/0	0,00	0/1	88,24	1/1	96,88
7	24239716	T	C	1903.03	0/0	0,00	0/1	84,21	1/1	96,77
7	150660065	C	A	3351.54	0/0	0,00	0/1	43,48	1/1	100,00
8	588396	G	A	3446.1	0/0	0,00	0/1	65,22	1/1	86,96
Y	11329952	A	C	241783.0	0/0	0,00	0/1	93,48	1/1	95,64
10	124522	T	A	1997.9	1/1	100,00	0/1	30,00	0/0	0,00

10	346611	G	A	6351.89	1/1	95,45	0/1	56,10	0/0	0,00
10	92730571	C	T	1066.18	1/1	100,00	0/1	86,67	0/0	0,00
10	92730574	C	T	1063.24	1/1	100,00	0/1	92,86	0/0	0,00
10	92730577	C	T	1192.59	1/1	100,00	0/1	88,24	0/0	0,00
10	92730580	C	T	1144.35	1/1	100,00	0/1	88,24	0/0	0,00
11	17711478	C	T	819.64	1/1	90,91	0/1	38,10	0/0	0,00
11	44523523	A	G	3045.36	1/1	92,86	0/1	57,89	0/0	0,00
11	44523545	G	A	3378.8	1/1	92,86	0/1	47,83	0/0	0,00
12	9953386	C	T	2765.37	1/1	100,00	0/1	92,86	0/0	0,00
13	102276380	T	C	2503.3	1/1	92,31	0/1	72,22	0/0	0,00
14	103302534	T	C	634.4	1/1	92,86	0/1	16,00	0/0	9,68
16	74854753	T	G	5410.26	1/1	100,00	0/1	57,14	0/0	0,00
17	76794698	T	G	4107.65	1/1	100,00	0/1	78,57	0/0	0,00
18	1540939	G	T	126.29	1/1	0,00	0/1	23,53	0/0	0,00
18	71247053	A	T	3225.85	1/1	97,14	0/1	55,00	0/0	0,00
2	47085051	A	T	1714.22	1/1	100,00	0/1	25,00	0/0	0,00
2	67792639	G	A	3657.94	1/1	85,71	0/1	31,58	0/0	0,00
20	3441131	C	T	4435.73	1/1	100,00	0/1	53,66	0/0	0,00
22	10722901	C	T	8764.77	1/1	94,12	0/1	91,43	0/0	0,00
22	10722902	A	G	8749.65	1/1	93,94	0/1	94,12	0/0	0,00
22	27001314	T	A	1155.25	1/1	92,31	0/1	56,25	0/0	0,00
3	75927982	T	C	2489.61	1/1	100,00	0/1	58,33	0/0	0,00
4	49646439	T	A	1871.32	1/1	50,00	0/1	55,56	0/0	0,00
4	57084787	G	A	2985.77	1/1	100,00	0/1	81,25	0/0	0,00
5	49602326	T	A	45483.0	1/1	98,13	0/1	80,26	0/0	0,00
7	156266503	C	A	5228.64	1/1	93,75	0/1	63,33	0/0	0,00
7	156266504	T	C	5228.64	1/1	93,75	0/1	64,52	0/0	0,00
