# Supplementary material for: Whole-genome sequencing of artificial single-nucleotide variants induced by DNA degradation in biological crime scene traces
Source: Int J Legal Med. 2022 Nov 10;137(1):33–45. doi: 10.1007/s00414-022-02911-0 (PMC9816238; doi:10.1007/s00414-022-02911-0)
Supplement: Supplementary file 3 — Supplementary file3 (PDF 1756 KB) [file 414_2022_2911_MOESM3_ESM.pdf]

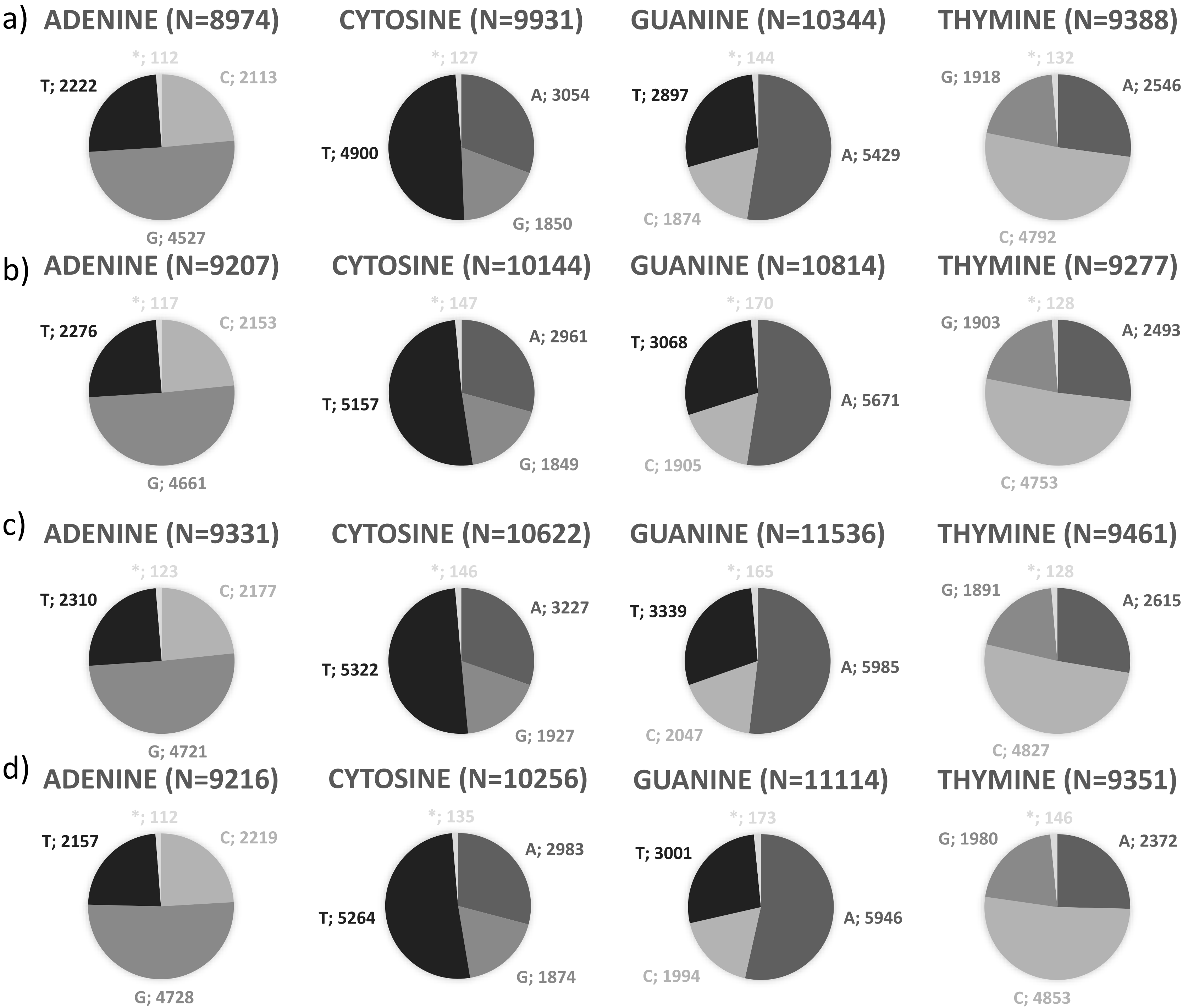


**day0=0/0; day22=0/1:**

a) *saliva humid,*
b) *saliva dry,*
c) *blood humid,*
d) *blood dry*

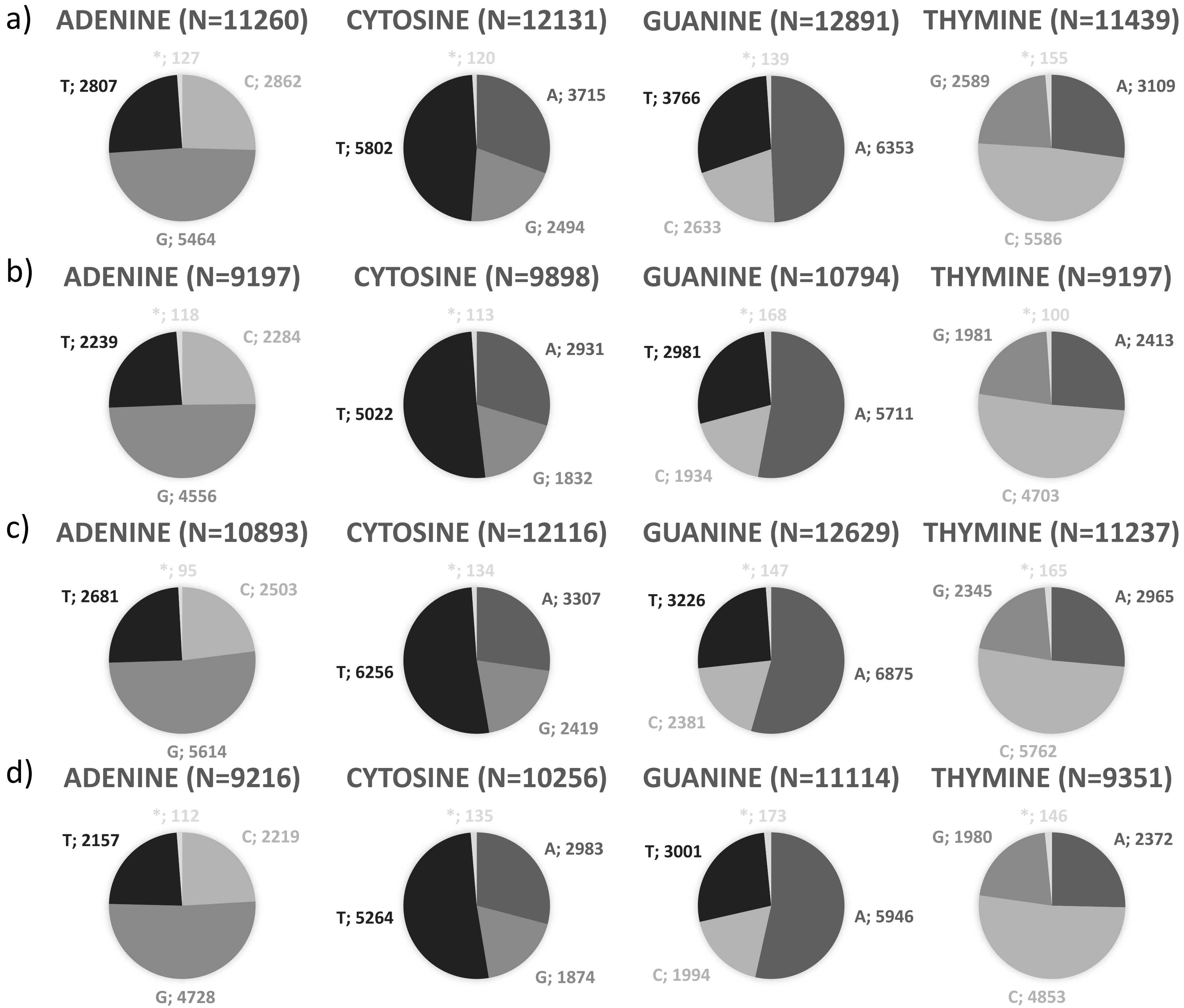


**day0=0/0; day92=0/1:**

a) *saliva humid,*
b) *saliva dry,*
c) *blood humid,*
d) *blood dry*

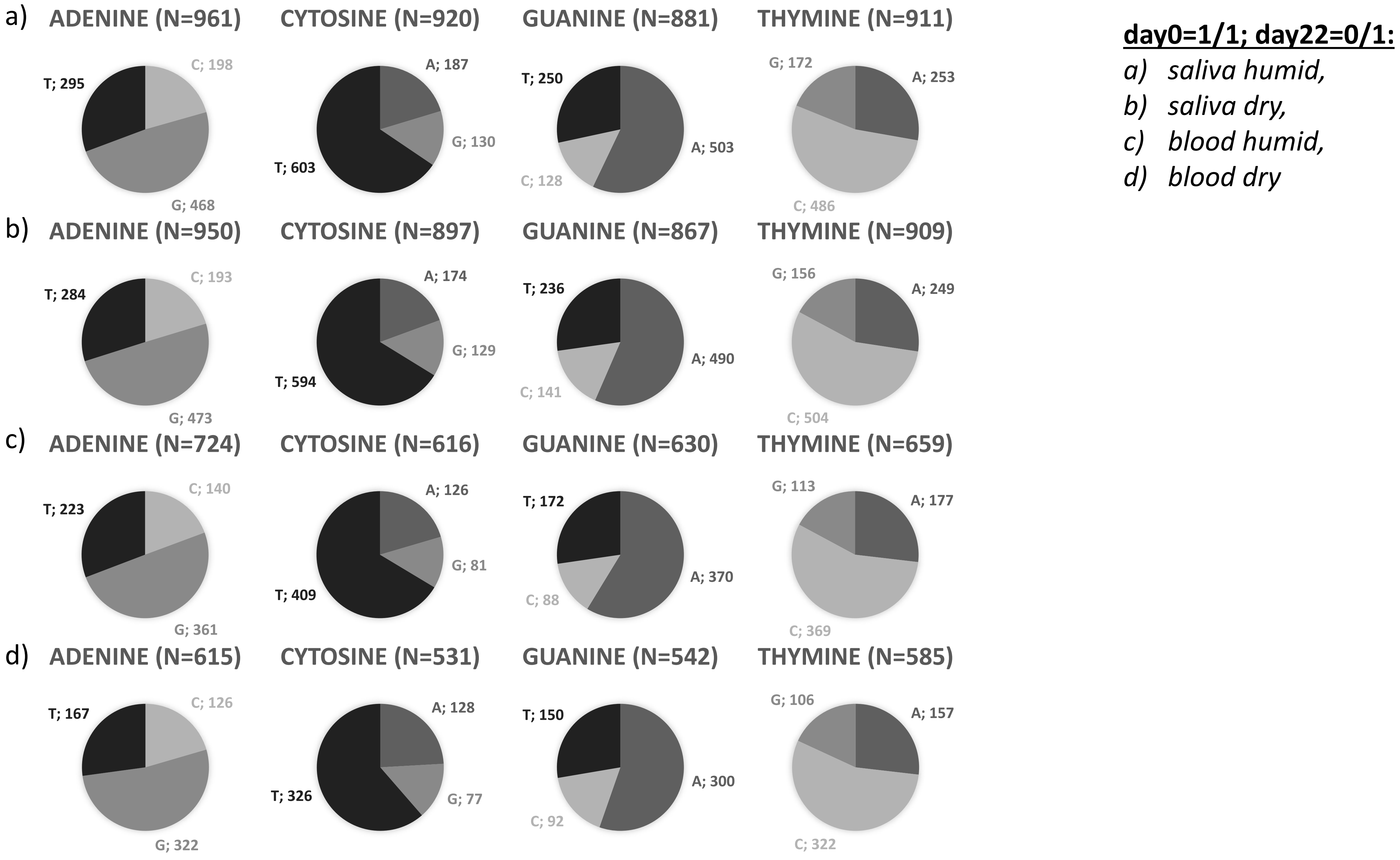
a)
ADENINE (N=961)
T; 295
C; 198
G; 468
CYTOSINE (N=920)
A; 187
G; 130
T; 603
GUANINE (N=881)
T; 250
A; 503
C; 128
THYMINE (N=911)
G; 172
A; 253
C; 486
b)
ADENINE (N=950)
T; 284
C; 193
G; 473
CYTOSINE (N=897)
A; 174
G; 129
T; 594
GUANINE (N=867)
T; 236
A; 490
C; 141
THYMINE (N=909)
G; 156
A; 249
C; 504
c)
ADENINE (N=724)
T; 223
C; 140
G; 361
CYTOSINE (N=616)
A; 126
G; 81
T; 409
GUANINE (N=630)
T; 172
A; 370
C; 88
THYMINE (N=659)
G; 113
A; 177
C; 369
d)
ADENINE (N=615)
T; 167
C; 126
G; 322
CYTOSINE (N=531)
A; 128
G; 77
T; 326
GUANINE (N=542)
T; 150
A; 300
C; 92
THYMINE (N=585)
G; 106
A; 157
C; 322
day0=1/1; day22=0/1:
a) saliva humid,
b) saliva dry,
c) blood humid,
d) blood dry

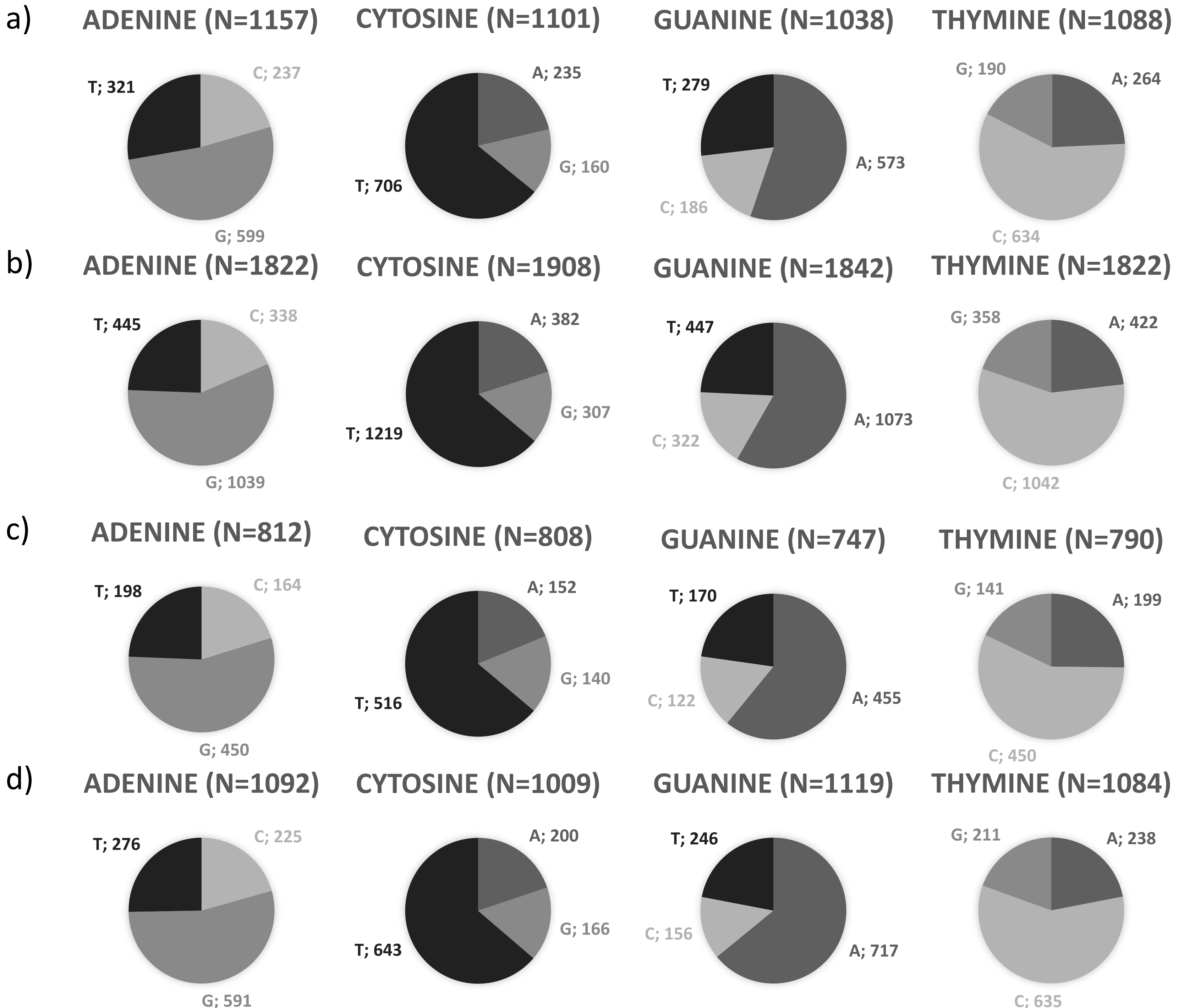
a)
ADENINE (N=1157)
T; 321
C; 237
G; 599
CYTOSINE (N=1101)
A; 235
G; 160
T; 706
GUANINE (N=1038)
T; 279
A; 573
C; 186
THYMINE (N=1088)
G; 190
A; 264
C; 634
b)
ADENINE (N=1822)
T; 445
C; 338
G; 1039
CYTOSINE (N=1908)
A; 382
G; 307
T; 1219
GUANINE (N=1842)
T; 447
A; 1073
C; 322
THYMINE (N=1822)
G; 358
A; 422
C; 1042
c)
ADENINE (N=812)
T; 198
C; 164
G; 450
CYTOSINE (N=808)
A; 152
G; 140
T; 516
GUANINE (N=747)
T; 170
A; 455
C; 122
THYMINE (N=790)
G; 141
A; 199
C; 450
d)
ADENINE (N=1092)
T; 276
C; 225
G; 591
CYTOSINE (N=1009)
A; 200
G; 166
T; 643
GUANINE (N=1119)
T; 246
A; 717
C; 156
THYMINE (N=1084)
G; 211
A; 238
C; 635

**day0=1/1; day92=0/1:**
a) *saliva humid,*
b) *saliva dry,*
c) *blood humid,*
d) *blood dry*

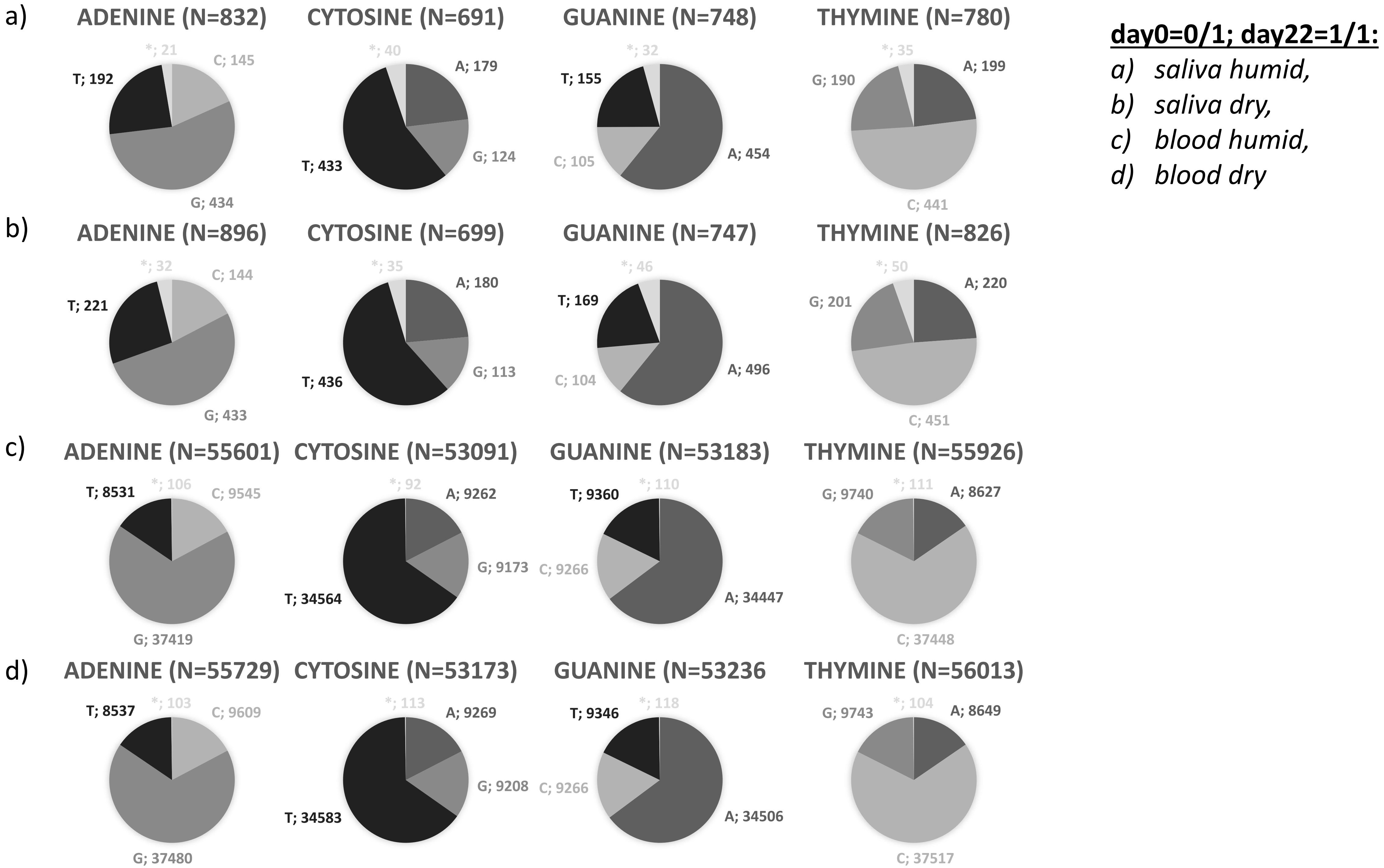
a)
ADENINE (N=832)
*; 21
C; 145
T; 192
G; 434
CYTOSINE (N=691)
*; 40
A; 179
G; 124
T; 433
GUANINE (N=748)
*; 32
T; 155
A; 454
C; 105
THYMINE (N=780)
*; 35
A; 199
G; 190
C; 441
b)
ADENINE (N=896)
*; 32
C; 144
T; 221
G; 433
CYTOSINE (N=699)
*; 35
A; 180
G; 113
T; 436
GUANINE (N=747)
*; 46
T; 169
A; 496
C; 104
THYMINE (N=826)
*; 50
A; 220
G; 201
C; 451
c)
ADENINE (N=55601)
*; 106
C; 9545
T; 8531
G; 37419
CYTOSINE (N=53091)
*; 92
A; 9262
G; 9173
T; 34564
GUANINE (N=53183)
*; 110
T; 9360
C; 9266
A; 34447
THYMINE (N=55926)
*; 111
A; 8627
G; 9740
C; 37448
d)
ADENINE (N=55729)
*; 103
C; 9609
T; 8537
G; 37480
CYTOSINE (N=53173)
*; 113
A; 9269
G; 9208
T; 34583
GUANINE (N=53236
*; 118
T; 9346
C; 9266
A; 34506
THYMINE (N=56013)
*; 104
A; 8649
G; 9743
C; 37517
day0=0/1; day22=1/1:
a) saliva humid,
b) saliva dry,
c) blood humid,
d) blood dry

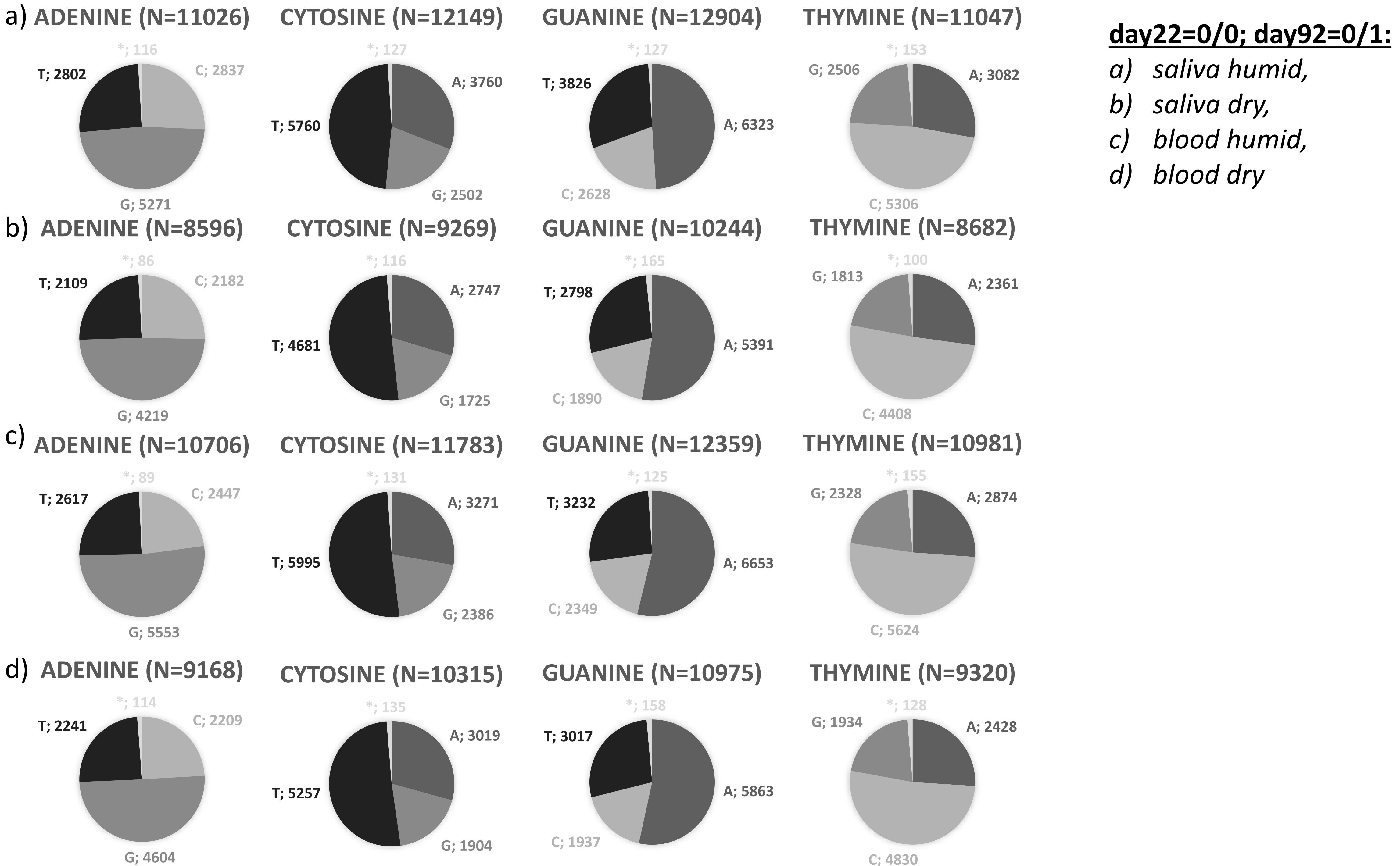
a) ADENINE (N=11026)
*; 116
C; 2837
T; 2802
G; 5271
CYTOSINE (N=12149)
*; 127
A; 3760
T; 5760
G; 2502
GUANINE (N=12904)
*; 127
T; 3826
A; 6323
C; 2628
THYMINE (N=11047)
*; 153
G; 2506
A; 3082
C; 5306
b) ADENINE (N=8596)
*; 86
T; 2109
C; 2182
G; 4219
CYTOSINE (N=9269)
*; 116
A; 2747
T; 4681
G; 1725
GUANINE (N=10244)
*; 165
T; 2798
A; 5391
C; 1890
THYMINE (N=8682)
*; 100
G; 1813
A; 2361
C; 4408
c) ADENINE (N=10706)
*; 89
T; 2617
C; 2447
G; 5553
CYTOSINE (N=11783)
*; 131
A; 3271
T; 5995
G; 2386
GUANINE (N=12359)
*; 125
T; 3232
A; 6653
C; 2349
THYMINE (N=10981)
*; 155
G; 2328
A; 2874
C; 5624
d) ADENINE (N=9168)
*; 114
T; 2241
C; 2209
G; 4604
CYTOSINE (N=10315)
*; 135
A; 3019
T; 5257
G; 1904
GUANINE (N=10975)
*; 158
T; 3017
A; 5863
C; 1937
THYMINE (N=9320)
*; 128
G; 1934
A; 2428
C; 4830
day22=0/0; day92=0/1:
a) saliva humid,
b) saliva dry,
c) blood humid,
d) blood dry

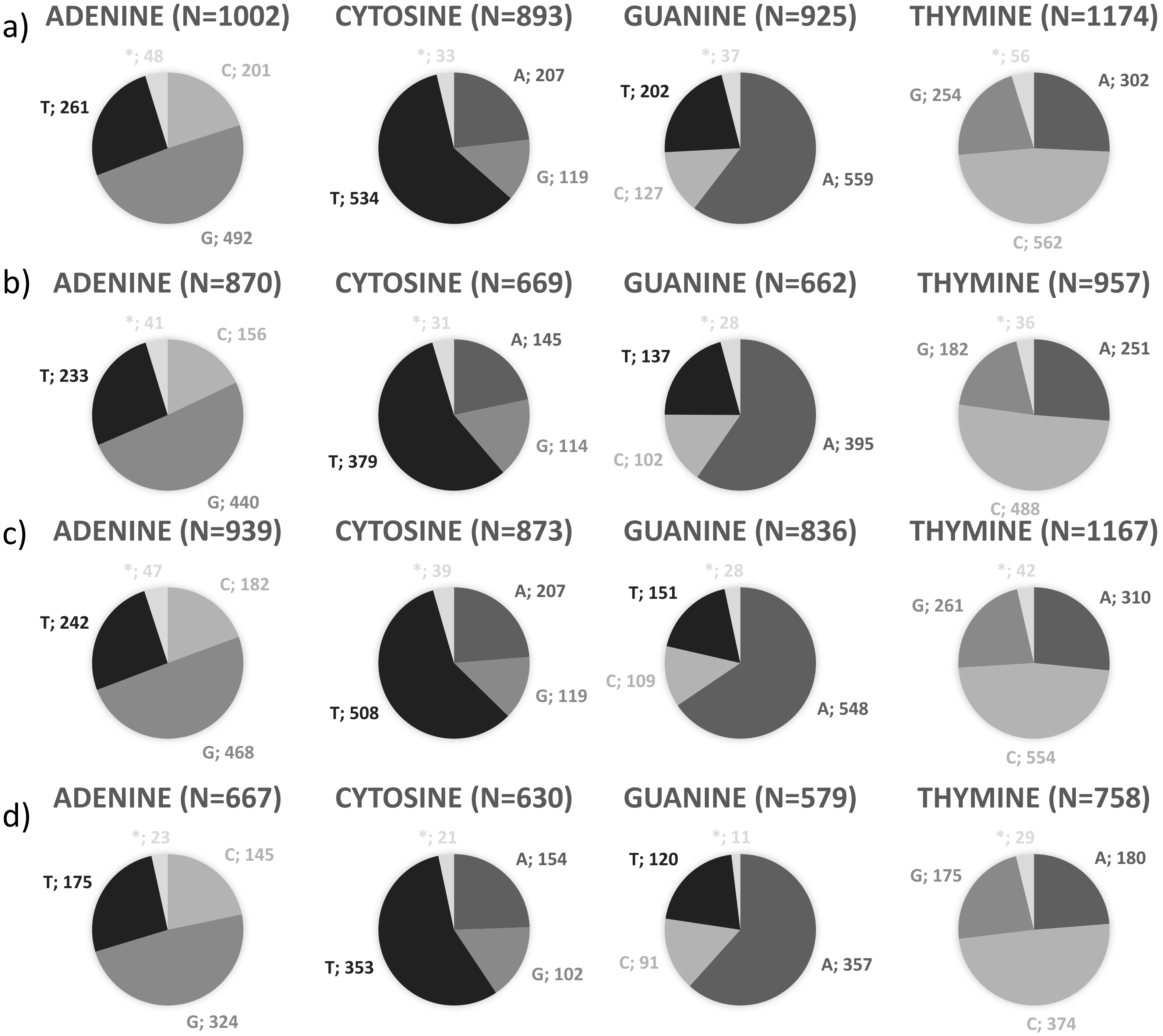


**day22=0/1; day92=1/1:**

a) *saliva humid,*
b) *saliva dry,*
c) *blood humid,*
d) *blood dry*

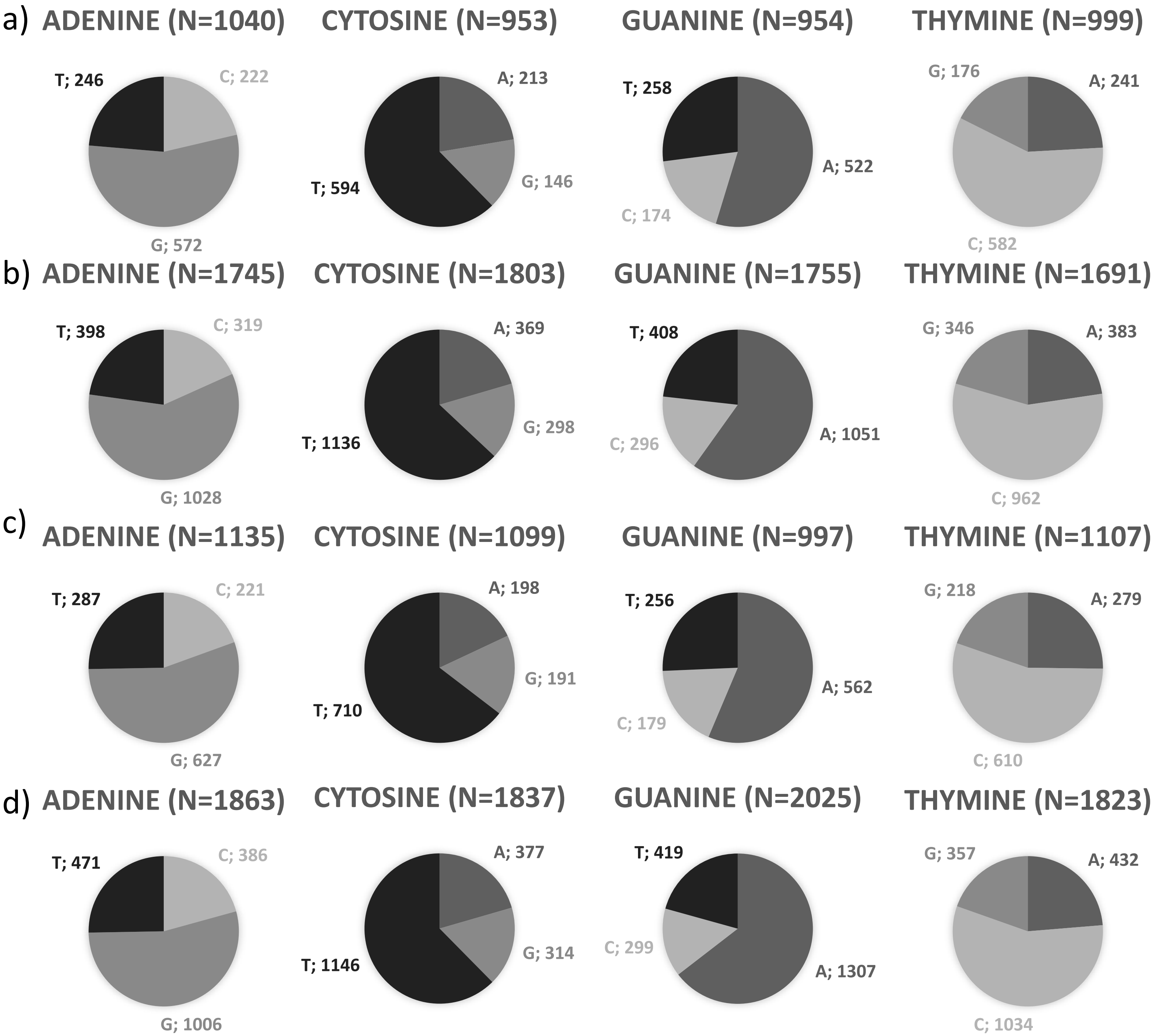


**day22=1/1; day92=0/1:**

*a) saliva humid,*
*b) saliva dry,*
*c) blood humid,*
*d) blood dry*

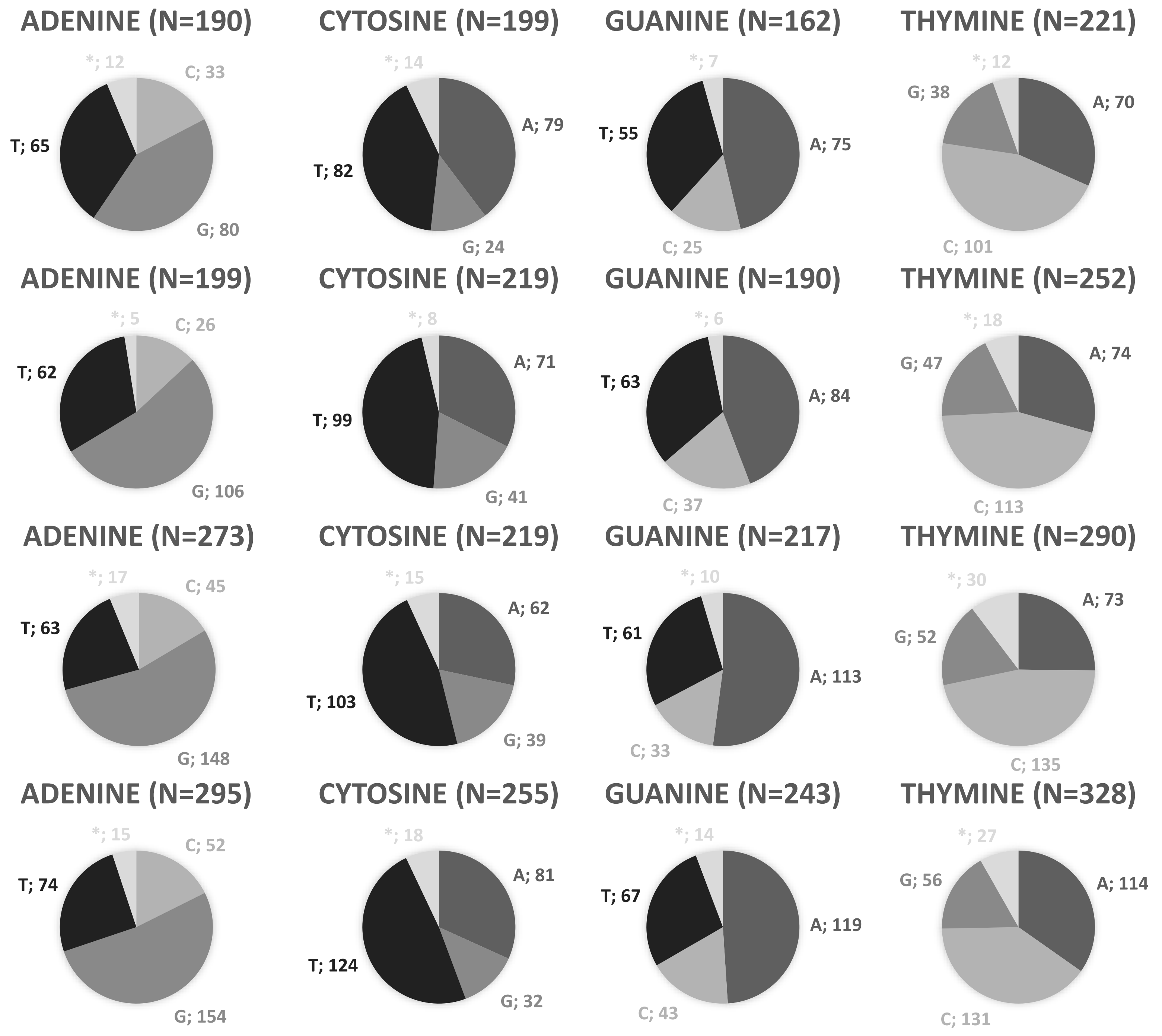


**<u>day0=0/0; day22=1/1:</u>**

a) *saliva humid,*
b) *saliva dry,*
c) *blood humid,*
d) *blood dry*

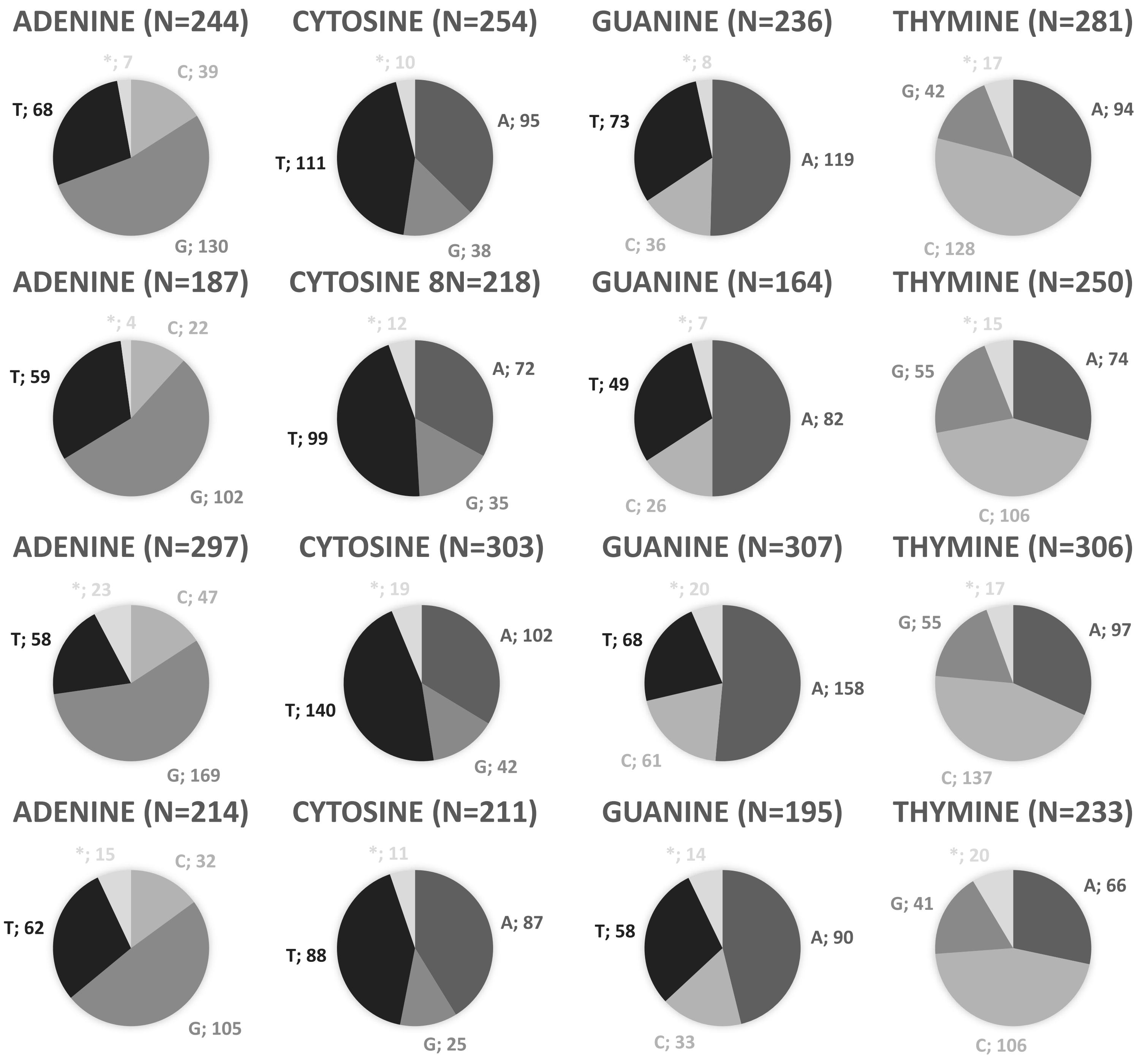


**<u>day0=0/0; day92=1/1:</u>**

a) *saliva humid,*
b) *saliva dry,*
c) *blood humid,*
d) *blood dry*

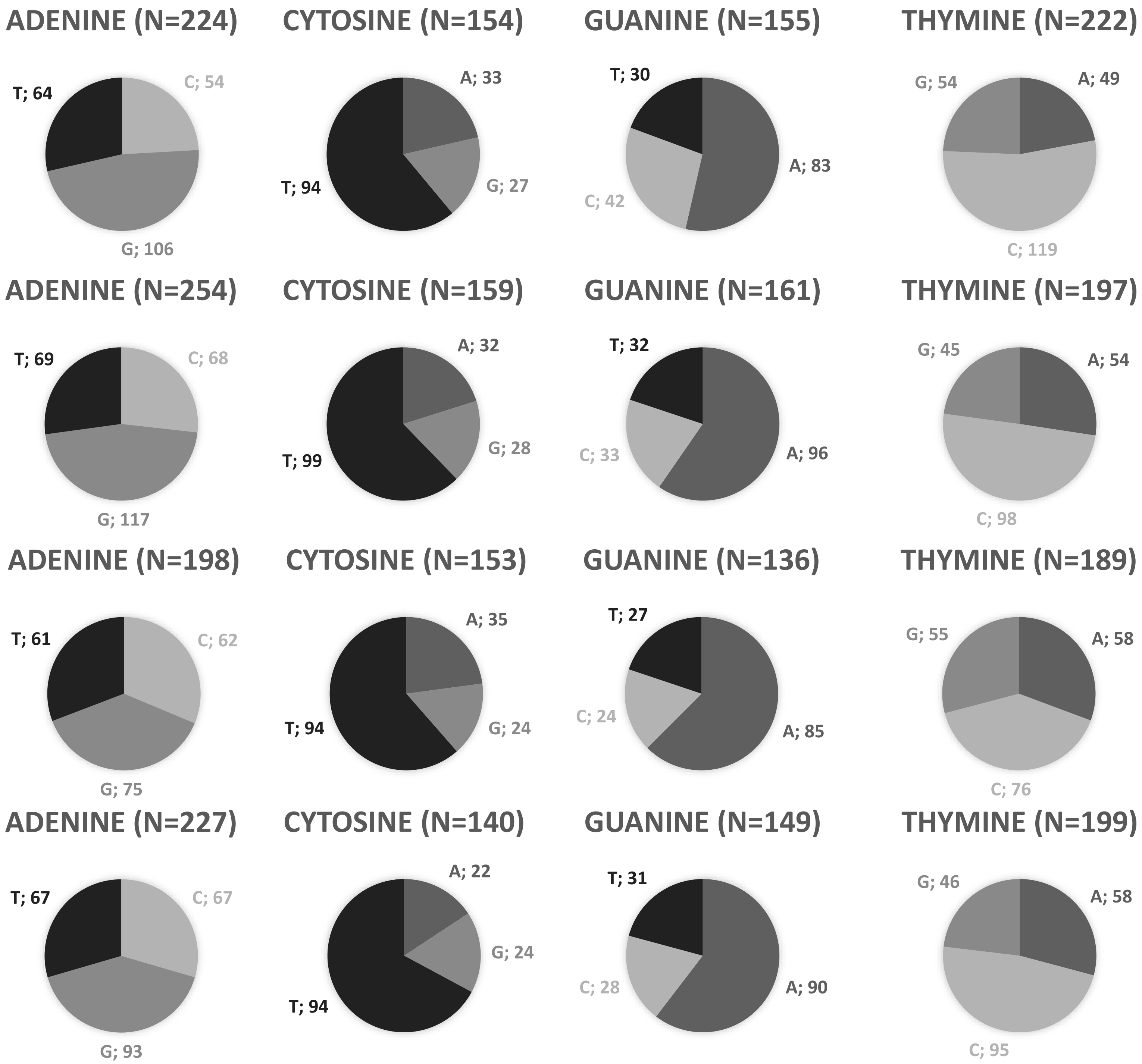


**<u>day0=1/1; day22=0/0:</u>**

a) *saliva humid,*
b) *saliva dry,*
c) *blood humid,*
d) *blood dry*

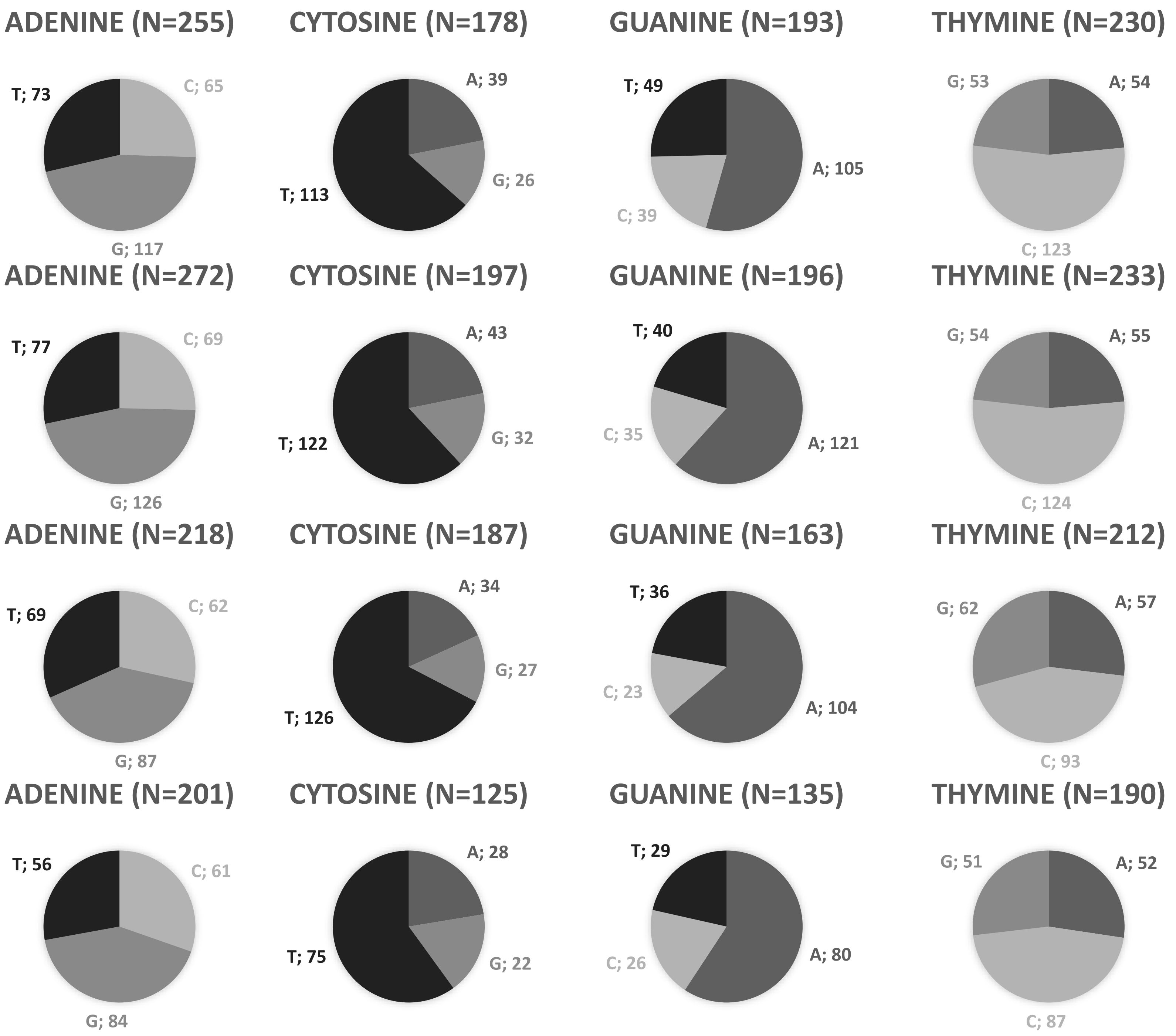


**day0=1/1; day92=0/0:**

a) *saliva humid,*
b) *saliva dry,*
c) *blood humid,*
d) *blood dry*
