# Supplementary material for: Whole-genome sequencing of artificial single-nucleotide variants induced by DNA degradation in biological crime scene traces
Source: Int J Legal Med. 2022 Nov 10;137(1):33–45. doi: 10.1007/s00414-022-02911-0 (PMC9816238; doi:10.1007/s00414-022-02911-0)
Supplement: Supplementary file 4 — Supplementary file4 (PDF 750 KB) [file 414_2022_2911_MOESM4_ESM.pdf]

blood humid										
CHROM	POS	REF	ALT	QUAL	GT 0 days	PRV 0 days	GT 22 days	PRV 22 days	GT 92 days	PRV 92 days
1	43904897	C	T	3894.69	0/0	0,00	0/1	31,58	0/1	52,94
11	21741151	C	T	612.99	0/0	11,11	0/1	21,05	0/1	53,85
11	76719528	C	T	4388.76	0/0	0,00	0/1	34,78	0/1	59,46
11	102731150	C	T	1749.96	0/0	4,55	0/1	28,57	0/1	66,67
12	107414722	C	T	2466.02	0/0	6,67	0/1	31,82	0/1	59,26
17	3031690	C	T	2699.75	0/0	0,00	0/1	34,62	0/1	61,54
17	78143099	C	T	4381.03	0/0	0,00	0/1	34,48	0/1	61,11
17	78143107	C	T	4242.91	0/0	0,00	0/1	33,33	0/1	55,00
19	1951819	C	T	1625.53	0/0	0,00	0/1	25,00	0/1	56,52
20	48510285	C	T	2524.98	0/0	0,00	0/1	32,43	0/1	53,19
22	11268337	C	T	2944.04	0/0	6,25	0/1	22,22	0/1	51,61
5	18096826	C	T	2890.02	0/0	0,00	0/1	28,57	0/1	53,33
6	32680262	C	T	1447.33	0/0	0,00	0/1	18,75	0/1	52,00
6	170265063	C	T	1183.1	0/0	0,00	0/1	22,22	0/1	62,96
7	116345921	C	T	1842.03	0/0	0,00	0/1	21,43	0/1	54,55
7	156035318	C	T	3902.76	0/0	0,00	0/1	25,93	0/1	53,57
7	158959904	C	T	3148.94	0/0	0,00	0/1	27,27	0/1	58,82
8	57204132	C	T	8030.76	0/0	0,00	0/1	31,82	0/1	59,70
9	113739953	C	T	1173.33	0/0	0,00	0/1	30,77	0/1	52,17
1	152534268	G	A	693.38	0/0	0,00	0/1	30,43	0/1	55,00
10	41843751	G	A	1484.02	0/0	9,09	0/1	20,00	0/1	51,28
10	42087342	G	A	2735.98	0/0	0,00	0/1	23,68	0/1	52,63
12	128783149	G	A	660.45	0/0	0,00	0/1	27,27	0/1	75,00
14	21887296	G	A	4424.41	0/0	0,00	0/1	34,48	0/1	61,29
14	74280670	G	A	474.43	0/0	14,29	0/1	29,41	0/1	66,67
17	21988482	G	A	952.47	0/0	0,00	0/1	20,69	0/1	54,55
2	740078	G	A	2009.48	0/0	0,00	0/1	33,33	0/1	65,12

2	25725731	G	A	4280.76	0/0	0,00	0/1	34,21	0/1	52,38
2	86545437	G	A	2742.92	0/0	0,00	0/1	31,25	0/1	51,28
5	1513773	G	A	3371.58	0/0	0,00	0/1	28,57	0/1	75,00
6	48507977	G	A	1569.53	0/0	0,00	0/1	30,00	0/1	61,54
6	60230561	G	A	6575.01	0/0	0,00	0/1	23,08	0/1	64,06
7	56764914	G	A	2646.97	0/0	7,14	0/1	25,00	0/1	66,67
7	56764920	G	A	2361.05	0/0	7,69	0/1	28,57	0/1	69,23
7	91888412	G	A	2528.76	0/0	0,00	0/1	33,33	0/1	52,17
8	57204104	G	A	7371.76	0/0	0,00	0/1	29,55	0/1	58,21
8	57204117	G	A	7540.76	0/0	0,00	0/1	31,11	0/1	59,09
8	139958904	G	A	1010.62	0/0	6,25	0/1	22,73	0/1	64,29
9	74346794	G	A	3380.98	0/0	0,00	0/1	29,17	0/1	57,14
KI270730.1	98453	G	A	2724.29	0/0	0,00	0/1	30,56	0/1	59,00
KI270756.1	4692	G	A	8588.76	0/0	0,00	0/1	28,38	0/1	52,13
1	168749522	A	G	4001.89	1/1	100,00	0/1	73,68	0/1	57,14
14	89333986	A	G	2918.07	1/1	84,62	0/1	65,00	0/1	52,63
14	103372214	A	G	4809.55	1/1	100,00	0/1	76,92	0/1	30,77
15	48578690	A	G	8419.99	1/1	100,00	0/1	76,00	0/1	61,54
16	58808204	A	G	2120.46	1/1	93,75	0/1	36,36	0/1	27,27
19	36827090	A	G	3476.45	1/1	93,33	0/1	57,14	0/1	50,00
20	833025	A	G	3121.3	1/1	100,00	0/1	64,71	0/1	56,52
3	85884979	A	G	5229.99	1/1	100,00	0/1	70,37	0/1	62,50
12	11883290	T	C	4588.11	1/1	94,74	0/1	75,00	0/1	63,64
12	11883293	T	C	4626.37	1/1	95,00	0/1	78,57	0/1	63,64
14	103372221	T	C	4956.41	1/1	100,00	0/1	73,33	0/1	23,53
20	6807051	T	C	6264.24	1/1	94,12	0/1	75,00	0/1	70,00
3	105402942	T	C	3526.19	1/1	90,00	0/1	76,47	0/1	60,71
3	195785374	T	C	10801.9	1/1	100,00	0/1	69,44	0/1	68,29
4	79062030	T	C	5360.62	1/1	90,00	0/1	76,19	0/1	70,97

4	110255848	T	C	2729.58	1/1	82,35	0/1	72,73	0/1	62,96
5	80548919	T	C	5757.85	1/1	100,00	0/1	73,68	0/1	60,00
5	80548921	T	C	6481.15	1/1	100,00	0/1	73,68	0/1	63,64
6	32552241	T	C	14504.0	1/1	96,30	0/1	68,89	0/1	60,42
6	32588210	T	C	13440.0	1/1	96,88	0/1	67,86	0/1	43,59
6	168611420	T	C	1480.45	1/1	94,12	0/1	45,45	0/1	40,91
7	31598404	T	C	4076.9	1/1	90,32	0/1	76,47	0/1	75,68
7	77008435	T	C	4336.88	1/1	100,00	0/1	76,92	0/1	61,11
8	89699534	T	C	5951.1	1/1	92,86	0/1	57,89	0/1	44,44
KI270442.1	162796	T	C	6650.99	1/1	96,43	0/1	27,03	0/1	20,24
KI270438.1	106744	T	C	446612.0	1/1	93,69	0/1	75,00	0/1	72,54
**blood dry**										
CHROM	POS	REF	ALT	QUAL	GT 0 days	PRV 0 days	GT 22 days	PRV 22 days	GT 92 days	PRV 92 days
12	128473363	C	T	5075.05	0/0	0,00	0/1	33,33	0/1	76,74
6	160851964	C	T	4493.76	0/0	0,00	0/1	29,17	0/1	54,76
11	76719528	C	T	4388.76	0/0	0,00	0/1	31,75	0/1	53,66
3	106073828	C	T	3601.76	0/0	0,00	0/1	24,53	0/1	57,14
5	176530309	C	T	3407.33	0/0	0,00	0/1	34,62	0/1	63,16
22	16321529	C	T	3332.33	0/0	0,00	0/1	31,58	0/1	53,85
11	2332600	C	T	2795.76	0/0	0,00	0/1	31,91	0/1	52,94
17	3031690	C	T	2699.75	0/0	0,00	0/1	27,50	0/1	60,87
6	32549273	C	T	2667.46	0/0	0,00	0/1	23,81	0/1	56,52
6	32587198	C	T	2290.75	0/0	0,00	0/1	19,23	0/1	52,63
19	3332267	C	T	1773.96	0/0	0,00	0/1	25,00	1/1	90,91
16	32562232	C	T	1671.04	0/0	0,00	0/1	34,78	0/1	56,25
X	125540459	C	T	1454.09	0/0	0,00	0/1	33,33	0/1	60,87
Y	6535086	G	A	8112.29	0/0	0,00	0/1	33,33	0/1	57,35
GL000225.1	7762	G	A	5672.71	0/0	0,00	0/1	22,58	0/1	64,71
5	59291735	G	A	4216.76	0/0	0,00	0/1	25,93	0/1	54,55

16	160828	G	A	3987.76	0/0	0,00	0/1	26,67	0/1	57,89
3	106073860	G	A	3865.76	0/0	0,00	0/1	24,07	0/1	58,54
1	121792430	G	A	2557.56	0/0	0,00	0/1	25,00	0/1	61,54
8	3022166	G	A	2420.91	0/0	0,00	0/1	31,03	0/1	58,82
1	220537597	T	C	4027.67	1/1	94,12	0/1	68,42	0/1	66,67
1	224210605	T	C	6106.15	1/1	96,88	0/1	69,23	0/1	68,75
1	247195316	T	C	2390.8	1/1	94,44	0/1	60,00	0/1	44,00
12	113716530	T	C	3289.29	1/1	100,00	0/1	75,00	0/1	70,00
14	56727330	T	C	4836.25	1/1	87,50	0/1	78,95	0/1	65,22
14	103372221	T	C	4956.41	1/1	100,00	0/1	58,33	0/1	54,55
14	104809555	T	C	3872.1	1/1	100,00	0/1	76,92	0/1	72,73
16	27143319	T	C	2283.35	1/1	45,00	0/1	58,82	0/1	40,00
17	52226338	T	C	6247.38	1/1	83,33	0/1	78,95	0/1	64,52
20	53643079	T	C	4514.46	1/1	83,33	0/1	44,12	0/1	40,00
3	105587336	T	C	2980.49	1/1	96,00	0/1	75,00	0/1	60,87
3	164537139	T	C	4601.92	1/1	95,24	0/1	73,33	0/1	63,89
5	71165963	T	C	2783.48	1/1	100,00	0/1	69,23	0/1	63,64
6	32552241	T	C	14504.0	1/1	96,30	0/1	72,73	0/1	57,63
KI270438.1	1148	T	C	76962.0	1/1	90,76	0/1	74,40	0/1	73,81
10	13692138	A	G	7252.8	1/1	100,00	0/1	77,78	0/1	66,67
10	56161455	A	G	3840.98	1/1	100,00	0/1	75,00	0/1	64,29
14	103372214	A	G	4809.55	1/1	100,00	0/1	63,64	0/1	52,17
18	11521560	A	G	1708.58	1/1	72,22	0/1	38,46	0/1	37,04
19	36827090	A	G	3476.45	1/1	93,33	0/1	71,43	0/1	42,31
20	833025	A	G	3121.3	1/1	100,00	0/1	59,26	0/1	53,85
8	124417079	A	G	7819.79	1/1	96,00	0/1	75,00	0/1	60,98
9	10476750	A	G	4902.83	1/1	92,86	0/1	63,33	0/1	55,17

saliva humid										
CHROM	POS	REF	ALT	QUAL	GT 0 days	PRV 0 days	GT 22 days	PRV 22 days	GT 92 days	PRV 92 days
1	158726686	C	T	3693.73	0/0	0,00	0/1	31,43	0/1	56,52
1	161281670	C	T	5267.76	0/0	0,00	0/1	31,71	0/1	51,35
1	161281686	C	T	5081.76	0/0	0,00	0/1	28,21	0/1	53,13
1	227698472	C	T	2343.16	0/0	11,11	0/1	27,27	0/1	57,14
1	229431534	C	T	4360.25	0/0	0,00	0/1	18,75	0/1	61,68
10	111574458	C	T	2893.76	0/0	0,00	0/1	30,56	0/1	51,85
12	12653350	C	T	1649.01	0/0	0,00	0/1	33,33	0/1	52,94
12	59860589	C	T	2785.31	0/0	0,00	0/1	30,00	0/1	68,75
16	19671819	C	T	4953.76	0/0	0,00	0/1	30,77	0/1	51,52
17	80665523	C	T	898.05	0/0	0,00	0/1	23,53	0/1	71,43
22	23947001	C	T	1052.05	0/0	0,00	0/1	20,00	0/1	52,94
4	140762703	C	T	1655.98	0/0	0,00	0/1	28,57	0/1	53,85
5	102779964	C	T	1932.76	0/0	0,00	0/1	23,81	0/1	52,94
5	175115104	C	T	4907.41	0/0	0,00	0/1	19,57	0/1	79,84
6	32525519	C	T	997.83	0/0	0,00	0/1	23,53	0/1	66,67
6	60234652	C	T	2065.18	0/0	3,33	0/1	31,25	0/1	68,00
Y	56834056	C	T	17563.3	0/0	0,00	0/1	26,08	0/1	51,49
KI270519.1	67309	C	T	10673.9	0/0	0,00	0/1	25,45	0/1	53,92
1	229431529	G	A	4294.25	0/0	0,00	0/1	18,75	0/1	60,95
14	89333961	G	A	3171.94	0/0	0,00	0/1	25,00	0/1	64,71
16	4484481	G	A	3653.76	0/0	0,00	0/1	20,69	0/1	54,55
17	21857649	G	A	8857.44	0/0	0,00	0/1	28,92	0/1	53,00
18	77061261	G	A	5137.84	0/0	0,00	0/1	32,26	0/1	60,00
2	17964542	G	A	1404.53	0/0	0,00	0/1	32,00	0/1	56,25
2	71618254	G	A	594.66	0/0	0,00	0/1	20,00	0/1	56,25
20	37679438	G	A	1298.19	0/0	10,00	0/1	33,33	0/1	55,56
21	37655257	G	A	3677.98	0/0	0,00	0/1	29,41	0/1	52,17

22	35491901	G	A	1332.49	0/0	0,00	0/1	31,25	0/1	62,50
5	175115110	G	A	5000.41	0/0	0,00	0/1	19,57	0/1	79,84
5	175115115	G	A	5028.41	0/0	0,00	0/1	19,57	0/1	79,20
8	41721686	G	A	1747.94	0/0	0,00	0/1	22,22	0/1	52,63
X	112160900	G	A	4755.76	0/0	0,00	0/1	30,77	0/1	61,29
KI270726.1	26363	G	A	1455.95	0/0	0,00	0/1	30,00	0/1	55,00
11	44509824	T	C	4184.57	1/1	90,91	0/1	68,42	0/1	25,00
22	38207442	T	C	3193.62	1/1	93,75	0/1	62,07	0/1	31,58
6	32511676	T	C	5596.76	1/1	100,00	0/1	51,22	0/1	22,22
6	32511735	T	C	6119.94	1/1	95,83	0/1	53,57	0/1	30,00
6	32573800	T	C	7472.32	1/1	100,00	0/1	51,11	0/1	31,25
1	17202425	A	G	2781.43	1/1	90,91	0/1	38,24	0/1	20,83
2	238908991	A	G	5674.6	1/1	96,77	0/0	46,15	0/1	33,85
3	128500975	A	G	1876.5	1/1	93,75	0/1	44,44	0/1	30,43
**saliva dry**										
CHROM	POS	REF	ALT	QUAL	GT 0 days	PRV 0 days	GT 22 days	PRV 22 days	GT 92 days	PRV 92 days
10	111574458	C	T	2893.76	0/0	0,00	0/1	23,33	0/1	51,72
13	113033373	C	T	1783.1	0/0	0,00	0/1	30,00	0/1	76,00
15	17022929	C	T	4455.53	0/0	0,00	0/1	20,00	0/1	62,77
16	48952007	C	T	4920.76	0/0	0,00	0/1	33,33	0/1	55,81
17	47131890	C	T	1650.9	0/0	0,00	0/1	30,77	0/1	69,23
18	63086836	C	T	3122.21	0/0	0,00	0/1	20,83	1/1	75,00
20	37603403	C	T	3266.25	0/0	0,00	0/1	32,00	0/1	52,63
21	45739055	C	T	1384.48	0/0	0,00	0/1	31,82	0/1	53,33
6	32513794	C	T	7242.76	0/0	0,00	0/1	22,58	0/1	58,49
6	46744323	C	T	1981.86	0/0	0,00	0/1	32,14	0/1	53,57
6	46744337	C	T	1883.78	0/0	0,00	0/1	29,63	0/1	54,17
6	46744349	C	T	1915.86	0/0	0,00	0/1	32,00	0/1	53,85
6	73602363	C	T	2874.76	0/0	0,00	0/1	32,35	0/1	55,88

7	158959904	C	T	3148.94	0/0	0,00	0/1	28,95	0/1	52,78
KI270722.1	107938	C	T	2048.11	0/0	0,00	0/1	33,33	0/1	57,58
KI270333.1	215	C	T	38188.5	0/0	0,00	0/1	28,57	0/1	91,33
13	113033327	G	A	1287.56	0/0	0,00	0/1	31,25	0/1	81,25
16	82032	G	A	1700.05	0/0	0,00	0/1	26,47	0/1	54,55
16	87975901	G	A	3491.33	0/0	0,00	0/1	30,95	0/1	54,55
4	3567911	G	A	3259.41	0/0	5,88	0/1	30,00	0/1	55,56
4	26215337	G	A	1679.76	0/0	0,00	0/1	26,32	0/1	54,55
4	119074970	G	A	3004.53	0/0	0,00	0/1	31,43	0/1	52,17
4	186821744	G	A	3457.53	0/0	0,00	0/1	31,82	0/1	52,17
7	393457	G	A	1525.76	0/0	0,00	0/1	28,57	0/1	60,00
8	143168375	G	A	1368.22	0/0	0,00	0/1	32,35	0/1	54,55
X	1350469	G	A	4998.01	0/0	0,00	0/1	29,73	0/1	56,36
KI270722.1	36048	G	A	4480.94	0/0	0,00	0/1	26,09	0/1	55,32
KI270438.1	47014	G	A	6841.09	0/0	0,00	0/1	30,43	0/1	62,69
